# Heterogeneity of perivascular astrocyte endfeet depending on vascular regions in the mouse brain

**DOI:** 10.1016/j.isci.2023.108010

**Published:** 2023-09-21

**Authors:** Takeshi Kameyama, Muneaki Miyata, Hajime Shiotani, Jun Adachi, Soichiro Kakuta, Yasuo Uchiyama, Kiyohito Mizutani, Yoshimi Takai

**Affiliations:** 1 Division of Pathogenetic Signaling, Department of Physiology and Cell Biology, Kobe University Graduate School of Medicine, Kobe 650-0047, Japan; 2 Department of Immunology and Parasitology, Graduate School of Medicine, Tokushima University, Tokushima 770-8503, Japan; 3 Division of Pathogenetic Signaling, Institute of Advanced Medical Sciences, Tokushima University, Tokushima 770-8503, Japan; 4 Laboratory of Proteomics for Drug Discovery, Center for Drug Design Research, National Institute of Biomedical Innovation, Health and Nutrition, Osaka 567-0085, Japan; 5 Laboratory of Clinical and Analytical Chemistry, Center for Drug Design Research, National Institute of Biomedical Innovation, Health and Nutrition, Osaka 567-0085, Japan; 6 Laboratory of Morphology and Image Analysis, Biomedical Research Core Facilities, Juntendo University Graduate School of Medicine, Tokyo 113-8421, Japan; 7 Department of Cellular Molecular Neuropathology, Juntendo University Graduate School of Medicine, Tokyo 113-8421, Japan

**Keywords:** Vascular anatomy, Immunology, Molecular neuroscience

## Abstract

Astrocytes interact with not only synapses but also brain blood vessels through perivascular astrocyte endfeet (PV-AEF) to form the neurovascular unit (NVU). However, PV-AEF components have not been fully identified. Here, we biochemically isolated blood vessels from mouse brain homogenates and purified PV-AEF. The purified PV-AEF were observed in different sizes, similar to PV-AEF on brain blood vessels. Mass spectrometry analysis identified 9,762 proteins in the purified PV-AEF, including cell adhesion molecules, nectin-2δ, Kirrel2, and podoplanin. Immunofluorescence microscopic analysis revealed that nectin-2δ and podoplanin were concentrated mainly in arteries/arterioles and veins/venules of the mouse brain, whereas Kirrel2 was mainly in arteries/arterioles. Nectin-2α/δ, Kirrel2, and podoplanin were preferentially observed in large sizes of the purified PV-AEF. Furthermore, Kirrel2 potentially has cell adhesion activity of cultured astrocytes. Collectively, these results indicate that PV-AEF have heterogeneity in sizes and molecular components, implying different roles of PV-AEF in NVU function depending on vascular regions.

## Highlights

• We biochemically isolated perivascular astrocyte endfeet from the mouse brain
• Mass spectrometry analysis identified novel cell adhesion molecules of PV-AEF
• The identified cell adhesion molecules localized at different vascular regions
• PV-AEF have heterogeneity in sizes and molecular components

## Graphical abstract

**Figure:**
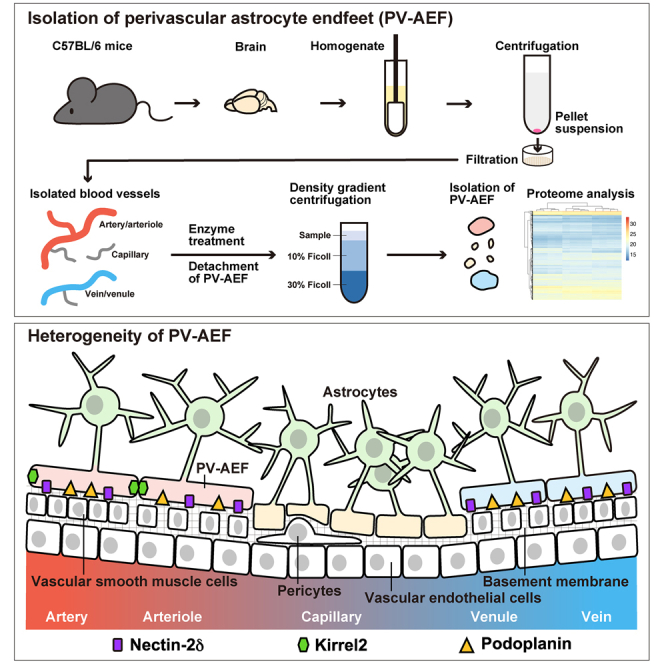

## Teaser

Vascular anatomy; Molecular neuroscience

## Introduction

Protoplasmic astrocytes, the predominant glial cell type in the brain, highly ramify by radiating from the cell soma primary branches that gradually divide into numerous fine terminal processes.^1^^,^^2^ They establish an interconnected network through terminal processes and further interact with numerous synapses through perisynaptic astrocyte processes to form tripartite synapses and with blood vessels through perivascular astrocyte endfeet (PV-AEF) to form neurovascular unit (NVU). PV-AEF constitute blood-brain barrier (BBB) cooperatively with vascular endothelial cells (VECs), mural cells including pericytes and vascular smooth muscle cells, fibroblasts, and basement membrane (BM) localizing between PV-AEF and VECs.^2^^,^^3^^,^^4^^,^^5^^,^^6^

During brain development, glutamatergic excitatory synapses are generated after astrocyte formation, whereas GABAergic inhibitory interneurons establish a functional network in the embryonic brain before astrocyte formation.^7^^,^^8^ In parallel with this, the blood vessel network is formed and established.^8^ Astrocytes start to ramify postnatally by first extending longer and less ramified processes, with spongiform process formation starting at the soma and extending centrifugally until all processes are equipped with fine terminal processes.^2^^,^^8^^,^^9^ At this stage, one process first approaches blood vessels to become PV-AEF to form NVU, and the remaining processes then approach many synapses of one neuron to form tripartite synapses and processes of neighboring astrocytes to establish an interconnected network.^1^^,^^2^^,^^8^^,^^9^

Neurons and blood vessels secrete many substances to astrocytes to regulate their differentiation and ramifications, whereas astrocytes also secrete many substances to neurons to regulate synapse formation, maturation, and function and to blood vessels to regulate angiogenesis, vessel relaxation and contraction, and BBB.^4^^,^^6^^,^^10^ Thus, neurons, astrocytes, and blood vessels mutually regulate their functions through many soluble factors. However, it remains unknown how astrocytes ramify and extend processes to blood vessels to form PV-AEF during the development of the brain.

Astrocytes are functionally polarized with perisynaptic astrocyte processes enriched with the glutamate transporters EAAT1/GLAST and EAAT2/GLT-1 and the inward rectifier K^+^ channel 4.1 (Kir4.1)^11^ and with PV-AEF enriched with aquaporin 4 (AQP4), glucose transporter 1, transient receptor potential vanilloid-related channel 4 (TRPV4), Kir4.1, megalencephalic leukoencephalopathy with subcortical cysts-1 (MLC1), and GlialCAM/hepaCAM.^12^^,^^13^^,^^14^^,^^15^^,^^16^^,^^17^^,^^18^^,^^19^ PV-AEF attach to the extracellular matrix proteins, such as laminin and agrin, in BM of blood vessels through the α-dystroglycan and β-dystroglycan complex. AQP4, TRPV4, and Kir4.1 localize by associating with β-dystroglycan through the peripheral membrane proteins α-dystrobrevin, β-dystrophin, and α-syntrophin.^2^^,^^15^ MLC1 and GlialCAM localize at the boundary between neighboring PV-AEF.^17^^,^^18^^,^^19^ We previously showed that nectin-2δ is concentrated in PV-AEF and localizes at the boundary between PV-AEF and BM of blood vessels.^20^^,^^21^ However, it still remains unknown how PV-AEF are enriched with many functional molecules during the development of the brain.

Recent vascular single-cell RNA transcriptome analysis revealed that the phenotypes of VECs and mural cells gradually change along the arteriovenous axis and that the transcriptional profiles of VECs and mural cells differ depending on vascular regions.^22^ Transcriptome analysis revealed that astrocyte heterogeneity is present across cortical layers in mice^23^ and that the expression profile of perivascular astrocytes is different from that of non-perivascular astrocytes in mice.^24^ However, the protein expression profiles of PV-AEF and their regional differences along blood vessels have not been fully understood.

In this study, we first biochemically isolated blood vessels from mouse brain homogenates, then isolated the purified PV-AEF from the isolated blood vessels and analyzed the molecular components of the purified PV-AEF using mass spectrometry. We identified 9,762 proteins in the purified PV-AEF. Among them, we focused on cell adhesion molecules (CAMs), nectin-2δ,^21^ Kirrel2/Neph3/filtrin,^25^^,^^26^^,^^27^ and podoplanin/aggrus/gp36/E11.^28^^,^^29^^,^^30^^,^^31^ We showed here that nectin-2δ and podoplanin localized mainly in PV-AEF of arteries/arterioles and veins/venules, but hardly in those of capillaries, whereas Kirrel2 localized mainly in PV-AEF of arteries/arterioles, but hardly in those of capillaries or veins/venules, in the mouse brain. These results indicate that PV-AEF have heterogeneity in sizes and molecular components, implying different roles of PV-AEF in BBB function depending on the regions of brain blood vessels.

## Results

### Isolation of blood vessels from mouse brain homogenates

**Figure 1:**
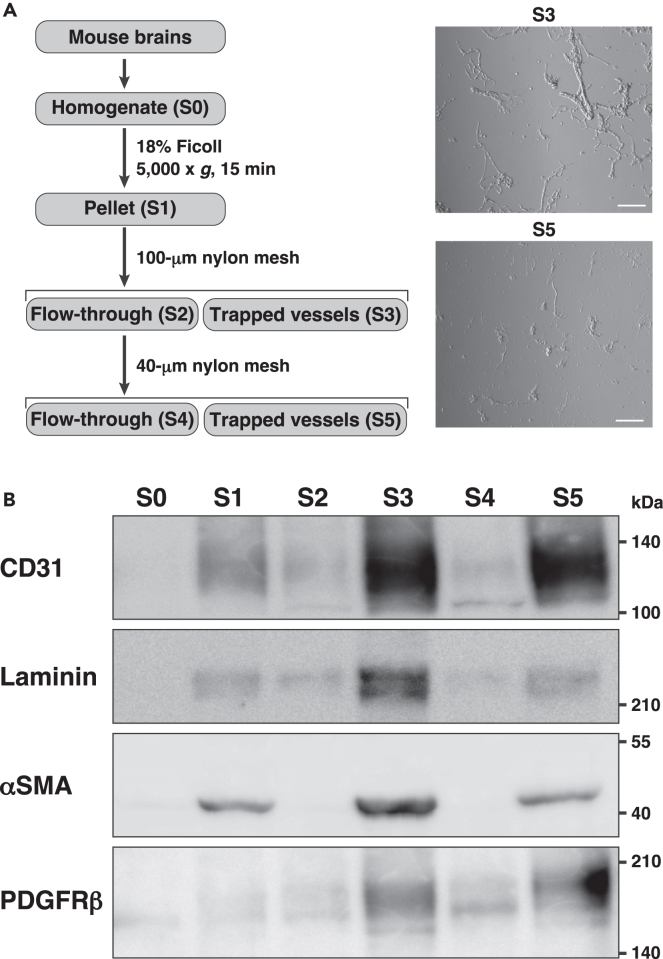
Isolation of blood vessels from the mouse brain (A) Outline procedure for the isolation of blood vessels from mouse brain homogenate. S3 and S5 were brain-blood-vessel-enriched fractions. Representative bright-field images of S3 and S5 fraction (right). S3 fraction contained large sizes of blood vessels compared with S5 fraction. Scale bars, 100 μm. (B) Immunoblot analysis of each fraction with the indicated Abs; 5 μg of total protein was loaded in each lane. CD31, VEC marker; Laminin, BM marker; αSMA, smooth muscle cell marker; and PDGFRβ, pericyte marker. As for laminin, laminin β1 and γ1 chains were detected. These images are representative of three independent experiments. See also Figure S1.

We firstly isolated blood vessels from the homogenates of eight-week-old mouse brains using previously published methods with slight modifications (Figure 1A).^32^^,^^33^ Fetal bovine serum (FBS) was added to the buffer, and all steps were carried out at 4°C to minimize proteolysis and protect against cell death. Microscopic analysis revealed that S3 and S5 fractions contained blood vessels with various lengths and sizes (Figure 1A). To analyze each fraction, western blot analysis was performed. The CD31 VEC marker signal was enriched in S3 and S5 fractions (Figure 1B). The alpha smooth muscle actin (αSMA) smooth muscle cell marker signal and the platelet-derived growth factor receptor beta (PDGFRβ) pericyte marker signal were also enriched in S3 and S5 fractions. In addition, the αSMA signal of S3 fraction was higher than that of S5. The laminin BM marker signal was also enriched in S3 and S5 fractions. The viability of vascular cells in the isolated blood vessels was then examined. The isolated blood vessels were calcein-AM-positive and propidium iodide-negative (Figure S1A). Neither the cleaved caspase-3 signal nor the TUNEL signal was observed in the isolated blood vessels (Figures S1B and S1C). These results indicate that the isolated structures are vital blood vessels containing various lengths and sizes of brain blood vessels.

**Figure 2:**
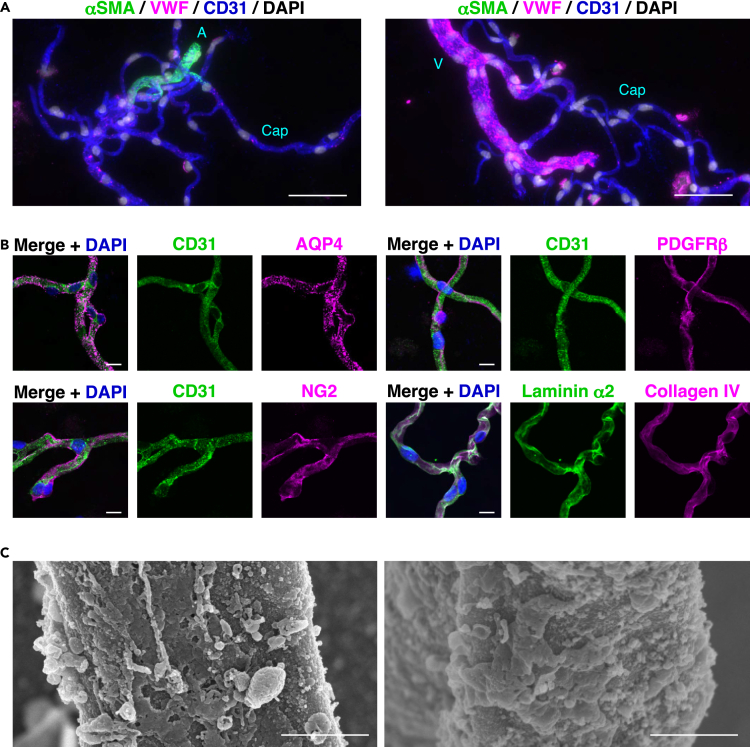
Characterization of the isolated brain blood vessels (A and B) Immunofluorescence images of the isolated blood vessels (mixture of S3 and S5 fractions) immunostained with the indicated Abs. Scale bars, 50 μm in (A) and 10 μm in (B). A, artery/arteriole; Cap, capillary; and V, vein/venule. (C) SEM analysis of the isolated blood vessels. These images are representative of two capillaries. Scale bars, 1 μm. These images are representative of three independent experiments. See also Figure S2.

Next, we characterized the details of the isolated blood vessels. Brain vasculatures consist of arteries, arterioles, capillaries, venules, and veins.^4^^,^^5^^,^^6^ To distinguish vascular regions, the specific marker of blood vessels was used. Consistent with a previous work,^22^ αSMA- and von Willebrand factor (VWF)-positive blood vessels were identified to be arteries and arterioles, whereas αSMA-negative and VWF-positive blood vessels were identified to be veins and venules. αSMA- and VWF-negative blood vessels were identified to be capillaries (Figure S2A). Using these markers, the vascular regions of the isolated blood vessels were identified. The isolated blood vessels contained αSMA- and VWF-positive regions, αSMA-negative and VWF-positive regions, and αSMA- and VWF-negative regions (Figure 2A). These results indicate that the isolated blood vessels consist of arteries/arterioles, capillaries, and veins/venules.

We next performed immunofluorescence microscopic analysis of the isolated blood vessels. The isolated blood vessels were immunostained not only with CD31 but also with the AQP4 astrocyte marker, the chondroitin sulfate proteoglycan NG2 and PDGFRβ pericyte markers, and the laminin α2 and collagen IV BM markers (Figure 2B); these blood vessels were partly immunostained with the PDGFRα fibroblast marker (Figure S2B), but not the myelin basic protein (MBP) oligodendrocyte or the ionized calcium-binding adapter molecule 1 (IBA1) microglia marker (Figures S2C and S2D). Scanning electron microscopic (SEM) analysis showed that various sizes of cell fragments and vesicles (length of major axis: >0.3 μm) were attached to the isolated blood vessels (Figures 2C and S2E). These results indicate that the isolated blood vessels consist of not only VECs, mural cells, fibroblasts, and BM but also PV-AEF, which may be torn off from astrocyte soma and processes during the isolation of blood vessels.

### Isolation of the purified PV-AEF with different sizes from the isolated blood vessels

**Figure 3:**
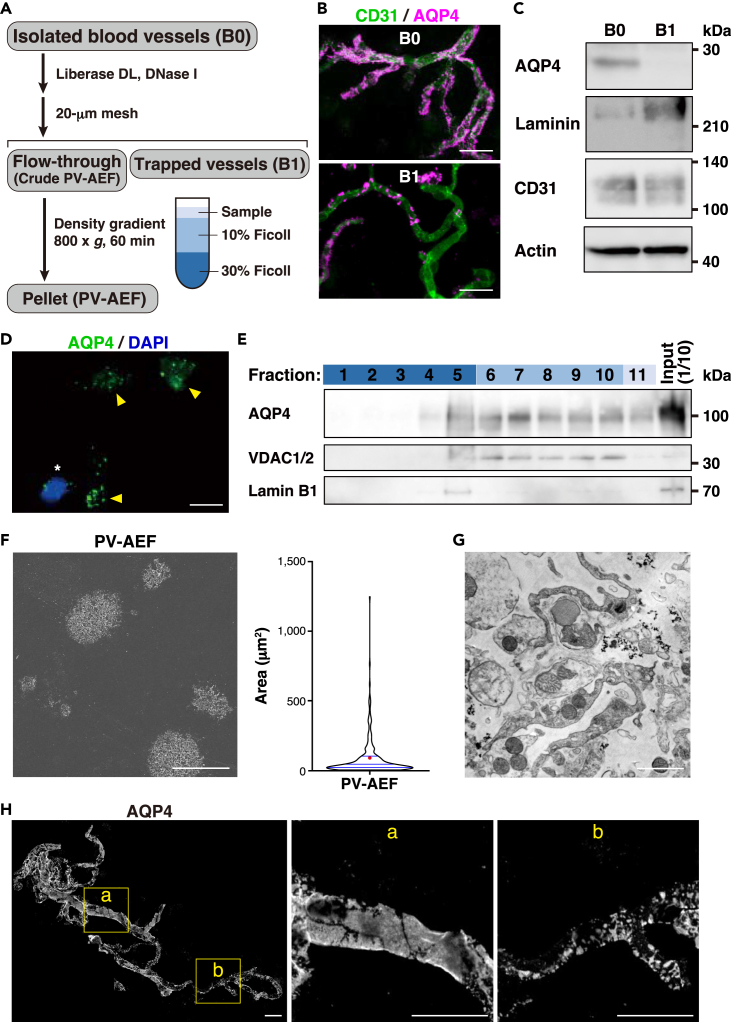
Isolation of the purified PV-AEF (A) Outline procedure for the isolation of the purified PV-AEF. B0, the isolated blood vessels (mixture of S3 and S5 fractions). B1, trapped vessels that detached PV-AEF. (B) Immunofluorescence images of B0 and B1 fractions immunostained with the indicated Abs. Scale bars, 50 μm. (C) Immunoblot analysis of B0 and B1 fractions with the indicated Abs. As for laminin, laminin β1 and γ1 chains were detected. (D) Immunofluorescence image of flow-through fraction. Arrowheads, PV-AEF identified by AQP4-positive and DAPI-negative cell fragments and vesicles. ∗, contaminated cells. Scale bar, 20 μm. (E) Density gradient fractionation of flow-through fraction. Equal volumes of each fraction were loaded on SDS-PAGE gels, and membranes were blotted with the indicated Abs. Input, flow-through fraction. In this experiment, AQP4 oligomer signal was observed. (F) SEM analysis of the purified PV-AEF. Scale bar, 20 μm. The area of the purified PV-AEF was shown in the violin plot (n = 270) (right). The red dot indicates average (84.1 μm^2^), and blue vertical lines indicate quartiles (median, 34.6 μm^2^). (G) TEM analysis of the purified PV-AEF. Scale bar, 1 μm. (H) Immunofluorescence images of the isolated mouse brain vessels immunostained with the anti-AQP4 Ab. a, b: enlargement of area outlined at left. a: large size of blood vessel (⌀, 12 μm); b: small size of blood vessels (⌀, 5 μm). Scale bars, 20 μm. These images are representative of three independent experiments. See also Figures S3 and S4.

We next isolated PV-AEF from the isolated blood vessels (Figure 3A). The isolated blood vessels (B0) were treated with Liberase DL and DNase I (Figure S3),^34^ followed by separation of PV-AEF-detached blood vessels (B1) and the crude PV-AEF by a 20-μm mesh filter. Immunofluorescence microscopic analysis revealed that the AQP4 signal in B1 was lower than that in B0 (Figure 3B), and western blot analysis showed that the AQP4 signal was mostly reduced in B1 (Figure 3C), suggesting that PV-AEF are detached from the isolated blood vessels. Indeed, in the crude PV-AEF, AQP4-positive and 4′,6-diamidino-2-phenylindole (DAPI)-negative cell fragments and vesicles were observed (Figure 3D). To remove contaminated cells from the crude PV-AEF, Ficoll density gradient centrifugation was performed. Western blot analysis revealed that the AQP4 signal was observed in Fractions 4–11, whereas the lamin B1 nuclear marker signal was observed in Fraction 5 (Figure 3E). Because PV-AEF contain elongated mitochondria,^35^ it was examined which fraction contained mitochondrial proteins. The VDAC1/2 mitochondria marker signal was observed in Fractions 6–10 (Figure 3E). SEM analysis also showed that BM structure was observed in B1 vessels (Figure S4A) and that Fractions 6–11 consisted of putative PV-AEF with various sizes ranging from 2.07 to 1,246.93 μm^2^ (length of major axis: 1.69–56.95 μm) (Figure 3F). Consistent with SEM analysis, various sizes of putative PV-AEF were also observed in transmission electron microscopic (TEM) analysis (Figures 3G and S4B). Presence of different sizes of putative PV-AEF observed in Fractions 6–11 was consistent with the different sizes of PV-AEF on brain blood vessels *in situ* as estimated by AQP4 immunostaining (Figure 3H) and *in vivo* as estimated by MLC1 immunostaining (Figure S4C). These results indicate that various sizes of PV-AEF are purified from the isolated brain blood vessels.

### Mass spectrometry analysis of the purified PV-AEF

We used Fractions 6–11 obtained from the Ficoll density gradient centrifugation as the purified PV-AEF and analyzed by high-resolution mass spectrometry to elucidate PV-AEF components. The isolated blood vessels (B0) and the crude PV-AEF fraction were used for the comparison of the purified PV-AEF. Four independent replicates, each of which was used from pooled tissues of different mice, were prepared. Firstly, to evaluate the intrasample variation of proteins, four samples from the isolated blood vessels (B0), the crude PV-AEF, and the purified PV-AEF were analyzed. A total of 11,062 proteins were identified in B0, and 9,432 proteins (85.3% of all B0 proteins identified) were common in all four samples (Figure S5A). A total of 10,506 proteins in the crude PV-AEF and 8,600 proteins (81.9% of all crude PV-AEF proteins identified) were common in all four samples (Figure S5B). A total of 9,762 proteins in the purified PV-AEF and 7,732 proteins (79.2% of all purified PV-AEF proteins identified) were common in all four samples (Figure S5C). The correlation coefficient of log_2_ intensity of identified proteins between two samples was higher than 0.95 (Figures S5A–S5C). Moreover, uniform manifold approximation and projection (UMAP) and hierarchical clustering analysis of log_2_ intensity for the 12 samples revealed a clear separation of the purified PV-AEF from the crude PV-AEF and B0 (Figures S5D and S5E). Thus, these results were convincing and used for further analysis.

**Figure 4:**
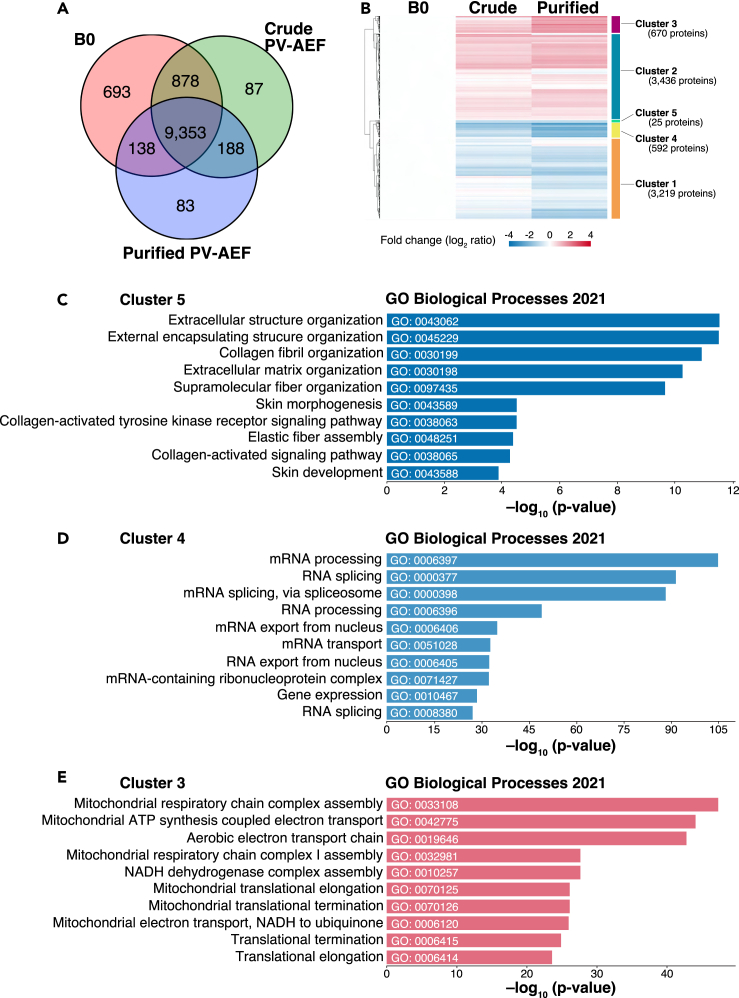
Mass spectrometry analysis of the purified PV-AEF (A) A Venn diagram of the numbers of identified proteins of each sample. B0, mixture of S3 and S5 fractions. (B) Heatmap and hierarchical clustering of significant differential expression of 8,432 proteins (ANOVA test). The color scale reflects the average of log_2_-fold changes in protein expression, compared with B0 (n = 4). The complete linkage method was used for hierarchical clustering. Crude, the crude PV-AEF; Purified, the purified PV-AEF. (C–E) Gene ontology enrichment analysis of biological processes for the indicated clusters. The top 10 enriched GO Biological Processes 2021 are shown. See also Figures S5–S7, Tables S1, S2, and S3.

A total of 11,420 proteins were identified among all samples (Figure 4A; Table S1); 9,762 proteins were identified in the purified PV-AEF, in which 9,353 proteins were common in all samples, 83 proteins were specific for the purified PV-AEF, and 188 proteins were observed in the purified PV-AEF and the crude PV-AEF (Figure 4A; Table S2). It was noted that these 83 proteins that were identified only in the purified PV-AEF were not identified in B0 or the crude PV-AEF, presumably because their concentrations were too low to be detected in B0 and the crude PV-AEF by the high-resolution mass spectrometry used here, indicating that these 83 proteins are highly enriched in the purified PV-AEF. Among 9,353 proteins that were identified to be common in all samples, 8,432 proteins showed significant differences in expression levels (ANOVA test, p < 0.05) (Table S3). Heatmap and hierarchical clustering analysis showed that these 8,432 proteins with significant differential expression proteins by ANOVA test were divided into five major clusters (Figure 4B). Cluster 4 and Cluster 5 had exclusively decreased proteins in the crude PV-AEF and the purified PV-AEF, and Cluster 1 also had decreased proteins in the purified PV-AEF, whereas Cluster 3 and Cluster 2 had exclusively increased proteins in the purified PV-AEF (Figure S6A). Gene ontology (GO) analysis revealed that Cluster 5 was majorly related to extracellular components (Figure 4C), and Cluster 4 was related to mRNA metabolism and gene expression (Figure 4D). Cluster 1 was related to post-translational modifications and transport (Figure S6B). Of note, Cluster 3 and Cluster 2 were related to mitochondria activity and translation processes (Figures 4E and S6C), consistent with the previous report showing that PV-AEF are enriched with mitochondria-related proteins.^36^ These results indicate that PV-AEF components are efficiently identified in this method.

### Characterization of three CAMs enriched in the purified PV-AEF

Nectin-2 is an immunoglobulin (Ig)-like CAM with three Ig-like domains and consists of two splicing isoforms, nectin-2α and nectin-2δ.^20^ We previously reported that nectin-2α was expressed in both cultured mouse neurons and astrocytes, whereas nectin-2δ was selectively expressed in the cultured mouse astrocytes and localized at the boundary between PV-AEF and BM of blood vessels in the brain.^21^ Furthermore, genetic ablation of *nectin-2* causes degeneration of PV-AEF and neurons in the cerebral cortex.^21^ Therefore, we firstly analyzed whether nectin-2 was identified in mass spectrometry analysis. Nectin-2 was identified in 9,353 proteins that were common in all samples (Figure 4A) and significantly enriched in the purified PV-AEF as nectin-2 belonged to Cluster 3 (Figures 4B and S7A). However, the localization of nectin-2δ in different vascular regions has not been clarified in the previous study.

To identify other novel CAMs of PV-AEF, we filtered the identified protein list by “cell adhesion” of GO biological processes. Three proteins were identified in 83 proteins specific for the purified PV-AEF (Figure 4A): IgLon5;Ntm, Itgb6, and Rgmb (Figure S7B). Single-cell RNA transcriptome analysis revealed that these three proteins were not preferentially expressed in astrocytes as analyzed by Betsholz dataset and Allen Brain Map. Nine proteins were identified in 188 proteins that were commonly observed in the purified PV-AEF and the crude PV-AEF, but not in B0 (Figure 4A): Bmp10, Cdh20, Cib1, Ephb1, Ephb1;Ephb2, Fzd1;Fzd2;Fzd7, Grid1;Grid2, Kirrel2, and Ptprt (Figures S7C and S7D). Among them, we focused on the Ig-like CAM Kirrel2,^25^^,^^26^^,^^27^ because single-cell RNA transcriptome analysis revealed that *Kirrel2* expression was predominantly observed in astrocytes (Figures S7E and S7F). Kirrel2 is one of the kin of irregular chiasm-1-like (Kirrel) family members, also known as nephrin-like family (Neph).^25^^,^^26^^,^^27^ The Kirrel family members are cell surface receptors and are involved in controlling axonal coalescence in the optic chiasm, olfactory bulb, and accessory olfactory bulb.^37^^,^^38^^,^^39^ *Kirrel2* expression of astrocytes was upregulated during mouse development (Figure S7G). However, the role of Kirrel2 in astrocytes remains unclear.

**Figure 5:**
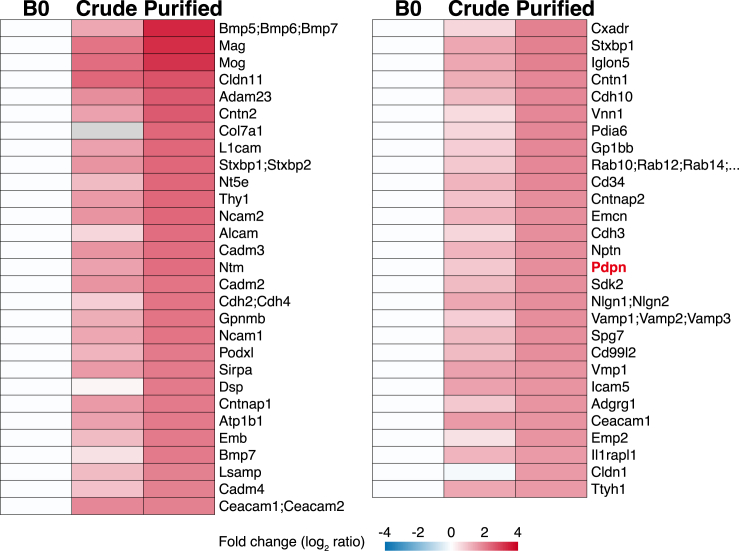
Mass spectrometry analysis of 57 CAMs Heatmap shows significantly upregulated 57 CAMs. The color scale reflects the average of log_2_-fold changes in protein expression compared with B0 (n = 4). See also Figure S8 and Table S3.

We further analyzed commonly identified 9,353 proteins (Figure 4A). Using 8,432 proteins that were significant differential expression by ANOVA test (Figure 4B; Table S3), CAMs significantly upregulated in the purified PV-AEF (p < 0.05 and fold change >3) were searched, and 57 proteins were identified (Figures 5 and S8A). Among these 57 proteins, we focused on mucin-type glycoprotein podoplanin/aggrus/gp36/E11,^28^^,^^29^^,^^30^^,^^31^ because single-cell RNA transcriptome analysis revealed that *Pdpn* was also preferentially expressed in astrocytes (Figures S8B and S8C). *Pdpn* was expressed in astrocytes during mouse development (Figure S8D). Podoplanin plays a crucial role in the development of the alveoli, heart, and lymphatic vascular system and the biology of immune cells through the interaction with the C-type lectin receptor CLEC-2.^31^ Although it was reported that cells expressing podoplanin are glial fibrillary acidic protein (GFAP)-positive astrocytes in the mouse brain,^40^ the detailed localization of podoplanin, especially in PV-AEF, remains unclear.

In addition, mRNA expression of these three CAMs was significantly enriched in perivascular astrocytes, compared with non-perivascular astrocytes as analyzed by McCarty dataset (Figure S8E).^24^ In this study, we further examined the detailed localization of nectin-2δ, Kirrel2, and podoplanin, especially in PV-AEF in the different vascular regions.

### *In vivo* localization of nectin-2**δ**, Kirrel2, and podoplanin in brain blood vessels

**Figure 6:**
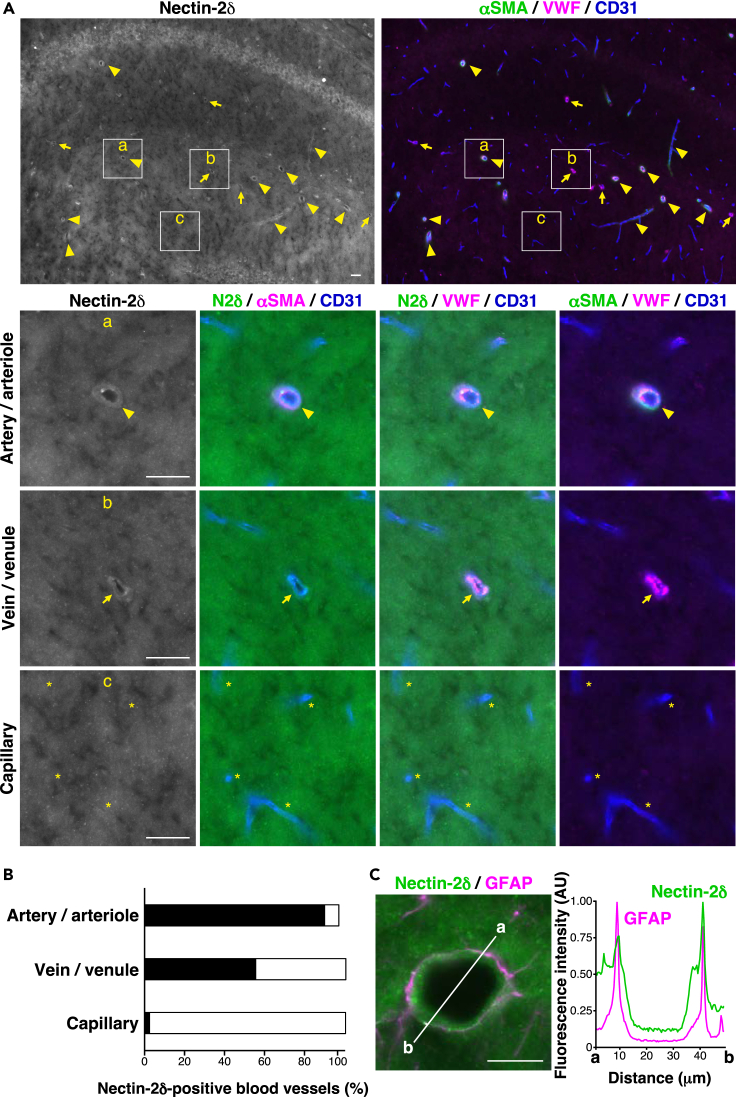
Localization of nectin-2**δ** in the mouse hippocampus (A) Localization of nectin-2δ (N2δ) in stratum lacunosum-moleculare of the hippocampus. a, artery/arteriole; b, vein/venule; and c, capillary. Arrowheads, arteries/arterioles; arrows, veins/venules; ∗, capillaries. Scale bars, 20 μm. (B) The ratio of nectin-2δ-positive blood vessels in the hippocampus is shown as a bar graph. (C) Co-localization of nectin-2δ and GFAP at artery/arteriole in stratum lacunosum-moleculare of the hippocampus. Scale bar, 20 μm. Line scan of fluorescence intensity of the white line (a–b). AU, arbitrary units. These images are representative of three independent experiments. See also Figures S9 and S12.

**Figure 7:**
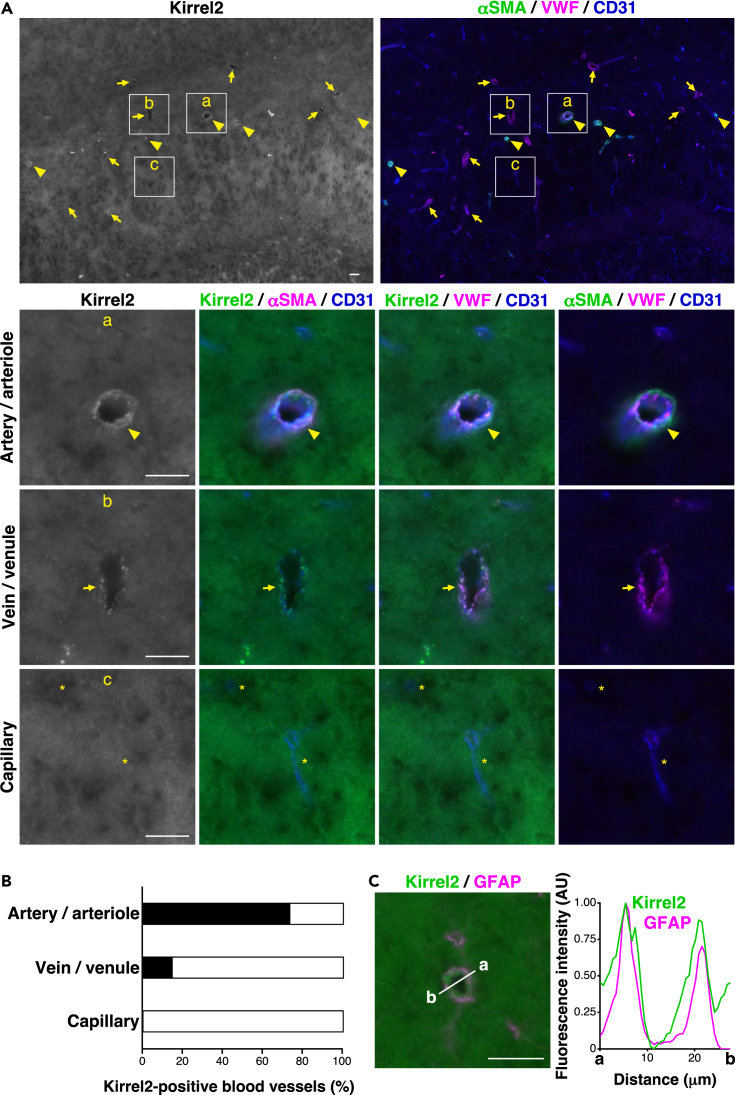
Localization of Kirrel2 in the mouse hippocampus (A) Localization of Kirrel2 in stratum lacunosum-moleculare of the hippocampus. a, artery/arteriole; b, vein/venule; and c, capillary. Arrowheads, arteries/arterioles; arrows, veins/venules; ∗, capillaries. Scale bars, 20 μm. (B) The ratio of Kirrel2-positive blood vessels in the hippocampus is shown as a bar graph. (C) Co-localization of Kirrel2 and GFAP at artery/arteriole in stratum lacunosum-moleculare of the hippocampus. Scale bars, 20 μm. Line scan of fluorescence intensity of the white line (a–b). AU, arbitrary units. These images are representative of three independent experiments. See also Figures S10 and S12.

**Figure 8:**
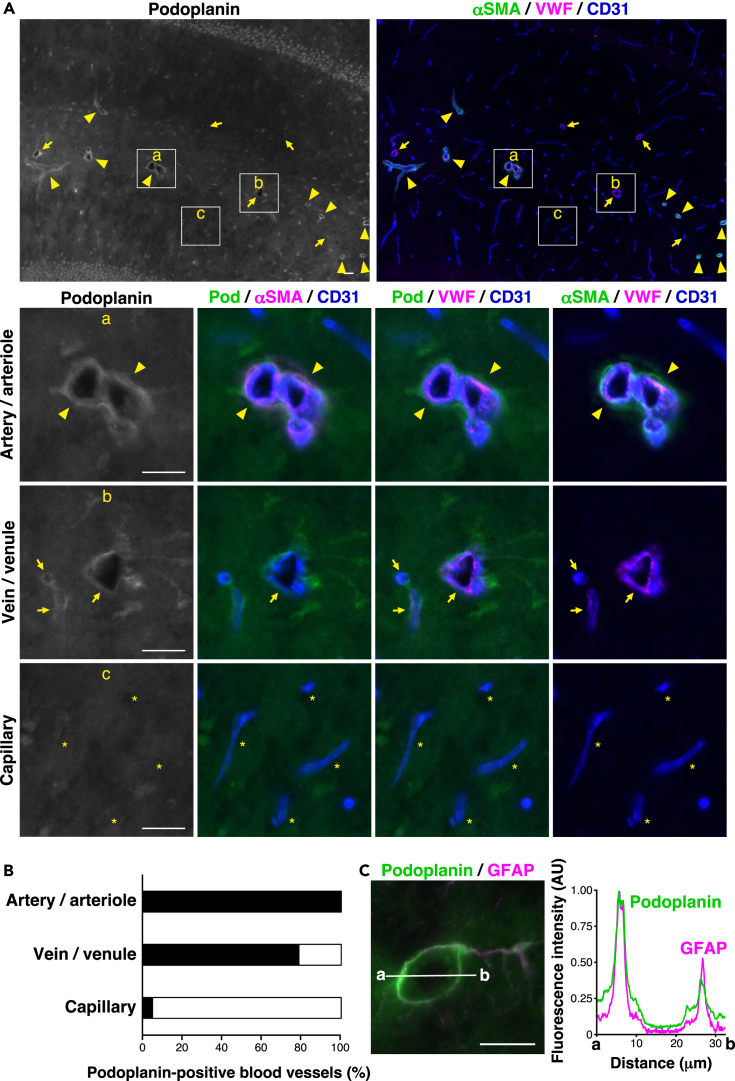
Localization of podoplanin in the mouse hippocampus (A) Localization of podoplanin (Pod) in stratum lacunosum-moleculare of the hippocampus. a, arteries/arterioles; b, veins/venules; and c, capillaries. Arrowheads, arteries/arterioles; arrows, veins/venules; ∗, capillaries. Scale bars, 20 μm. (B) The ratio of podoplanin-positive blood vessels in the hippocampus is shown as a bar graph. (C) Co-localization of podoplanin and GFAP at artery/arteriole in stratum lacunosum-moleculare of the hippocampus. Scale bar, 20 μm. Line scan of fluorescence intensity of the white line (a–b). AU, arbitrary units. These images are representative of three independent experiments. See also Figures S11 and S12.

We examined the localization of nectin-2δ, Kirrel2, and podoplanin in the mouse brain. The nectin-2δ signal was partly observed in αSMA- and VWF-positive blood vessels and αSMA-negative and VWF-positive blood vessels (Figures 6A, 6B, S9A, and S9B). On the contrary, the nectin-2δ signal was hardly observed in αSMA- and VWF-negative blood vessels (Figures 6A, 6B, S9A, and S9B). The nectin-2δ signal co-localized with the GFAP astrocyte marker signal (Figure 6C), indicating that nectin-2δ localizes at least in PV-AEF. The Kirrel2 signal was mostly observed in αSMA- and VWF-positive blood vessels and hardly observed in αSMA-negative and VWF-positive blood vessels or in αSMA- and VWF-negative blood vessels (Figures 7A, 7B, S10A, and S10B). The Kirrel2 signal co-localized with the GFAP signal (Figure 7C), indicating that Kirrel2 localizes at least in PV-AEF. The podoplanin signal was observed mostly in αSMA- and VWF-positive blood vessels and αSMA-negative and VWF-positive blood vessels and hardly observed in αSMA- and VWF-negative blood vessels (Figures 8A, 8B, S11A, and S11B). The podoplanin signal co-localized with the GFAP signal (Figure 8C), indicating that podoplanin localizes at least in PV-AEF. These results indicate that nectin-2δ and podoplanin localize mainly in arteries/arterioles and veins/venules, but hardly in capillaries, whereas Kirrel2 localizes mainly in arteries/arterioles, but hardly in capillaries or veins/venules. On the contrary, the AQP4 signal was observed in almost all the laminin α4-positive blood vessels (Figure S12). These results suggest that PV-AEF have different components depending on the different vascular regions.

### Localization of nectin-2**δ**, Kirrel2, and podoplanin in large sizes of PV-AEF

**Figure 9:**
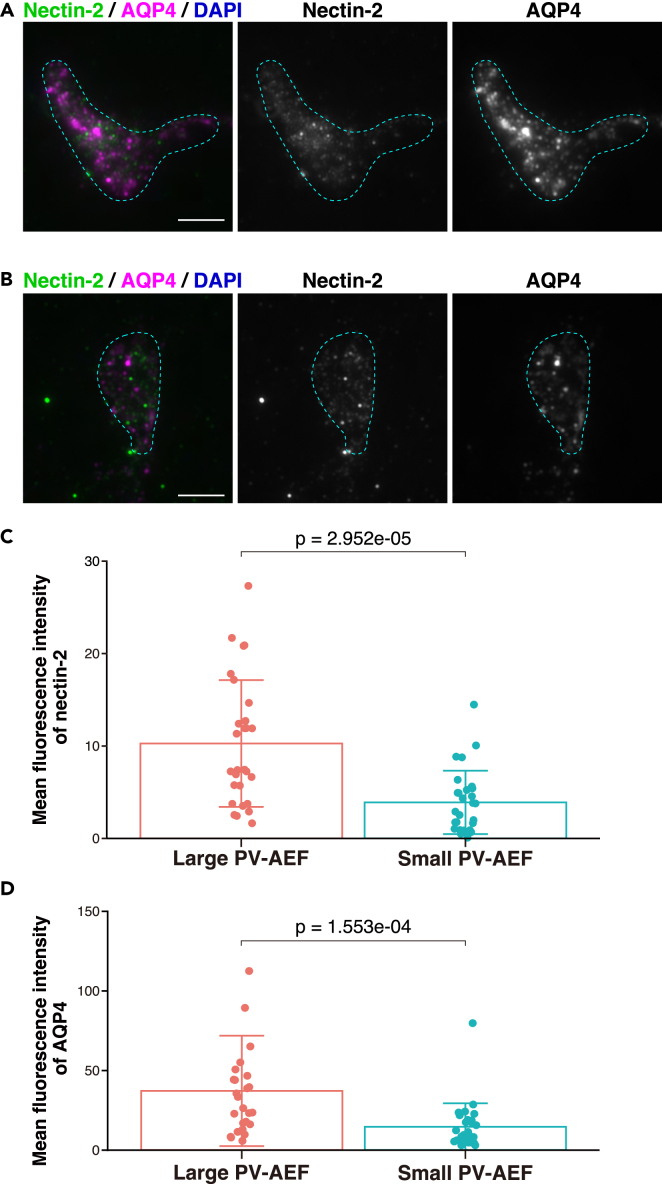
Localization of nectin-2α/δ in the purified PV-AEF (A and B) Immunofluorescence images of the purified PV-AEF immunostained with the indicated Abs. The anti-nectin-2 Ab, which recognizes nectin-2α/δ, was used. The purified PV-AEF were identified by AQP4-positive and DAPI-negative cell fragments and vesicles. The areas encircled by the cyan dotted line indicate the areas of the purified PV-AEF. Scale bars, 5 μm. (C and D) Mean fluorescence intensity of nectin-2 and AQP4 was shown in the bar graphs. The area of PV-AEF was measured by the AQP4 signal and divided into two groups according to the median value. Data are presented as mean and SD (n = 28, 28), and dot shows each data. The Mann-Whitney U test was performed. These images are representative of three independent experiments.

**Figure 10:**
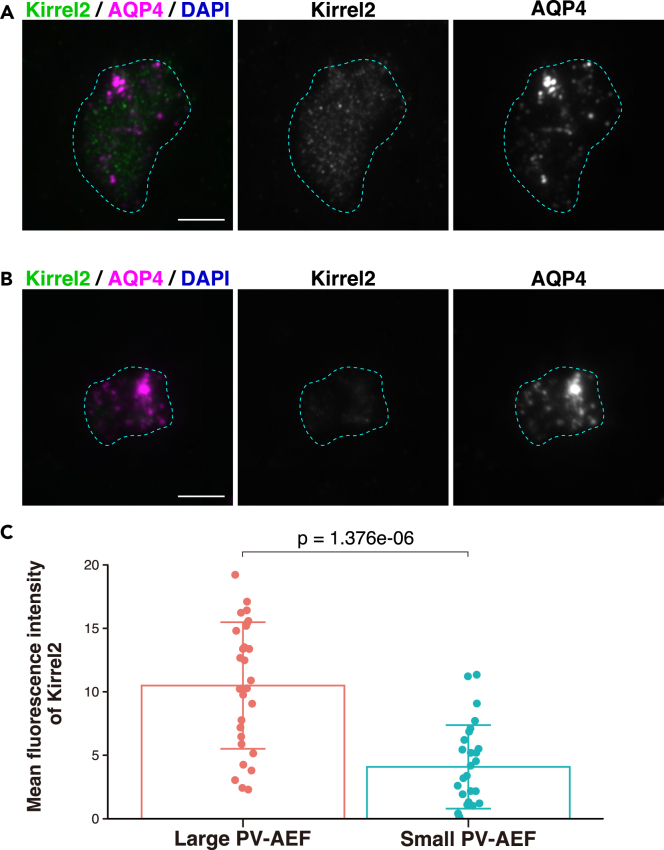
Localization of Kirrel2 in the purified PV-AEF (A and B) Immunofluorescence images of the purified PV-AEF immunostained with the indicated Abs. The purified PV-AEF were identified by AQP4-positive and DAPI-negative cell fragments and vesicles. The areas encircled by the cyan dotted line indicate the areas of the purified PV-AEF. Scale bars, 5 μm. (C) Mean fluorescence intensity of Kirrel2 was shown in the bar graphs. The area of PV-AEF was measured by the AQP4 signal and divided into two groups according to the median value. Data are presented as mean and SD (n = 28, 28), and dot shows each data. The Mann-Whitney U test was performed. These images are representative of three independent experiments.

**Figure 11:**
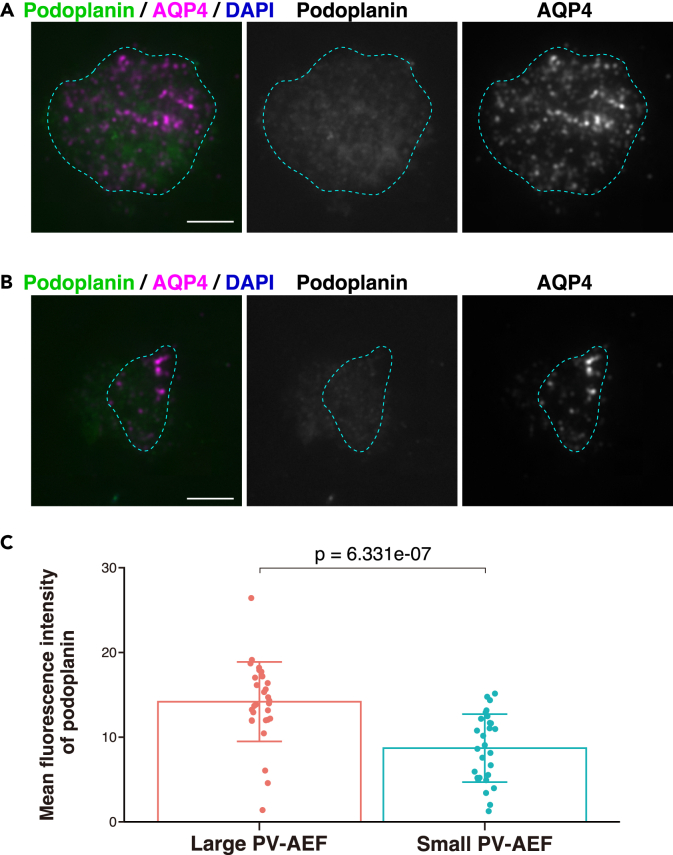
Localization of podoplanin in the purified PV-AEF (A and B) Immunofluorescence images of the purified PV-AEF immunostained with the indicated Abs. The purified PV-AEF were identified by AQP4-positive and DAPI-negative cell fragments and vesicles. The areas encircled by the cyan dotted line indicate the areas of the purified PV-AEF. Scale bars, 5 μm. (C) Mean fluorescence intensity of podoplanin was shown in the bar graphs. The area of PV-AEF was measured by the AQP4 signal and divided into two groups according to the median value. Data are presented as mean and SD (n = 31, 30), and dot shows each data. The Mann-Whitney U test was performed. These images are representative of three independent experiments.

We next assessed nectin-2δ, Kirrel2, and podoplanin expression in the purified PV-AEF. The clear nectin-2α/δ signal was observed in part of the purified PV-AEF (Figure 9A), and the faint signal was observed in another part of the purified PV-AEF (Figure 9B). As the purified PV-AEF showed various sizes (Figures 3F, 3H, and S4C), it was examined whether expression levels of nectin-2α/δ are different depending on the sizes of the purified PV-AEF. The purified PV-AEF were divided into two groups according to the median value of the purified PV-AEF area detected by AQP4 immunostaining and defined as large and small sizes of PV-AEF samples. The mean fluorescence intensity of nectin-2α/δ and AQP4 in large sizes of PV-AEF was significantly higher than that of small sizes of PV-AEF (Figures 9C and 9D). The clear Kirrel2 signal was observed in part of the purified PV-AEF (Figure 10A), and the faint signal was observed in another part of the purified PV-AEF (Figure 10B). The mean fluorescence intensity of Kirrel2 in large sizes of PV-AEF was significantly higher than that of small sizes of PV-AEF (Figure 10C). The clear podoplanin signal was observed in part of the purified PV-AEF (Figure 11A), and the faint signal was observed in another part of the purified PV-AEF (Figure 11B). The mean fluorescence intensity of podoplanin in large sizes of PV-AEF was significantly higher than that of small sizes of PV-AEF (Figure 11C). These results were consistent with the *in vivo* observations that nectin-2δ, Kirrel2, and podoplanin localized in arteries/arterioles and/or veins/venules (Figures 6, 7, 8, and S9–S11), where the sizes of PV-AEF were larger than those of capillaries (Figures 3H and S4C).^18^ Collectively, these results indicate that nectin-2α/δ, Kirrel2, and podoplanin preferentially localize in large sizes of PV-AEF.

### Adhesion activity of Kirrel2 in cultured astrocytes

**Figure 12:**
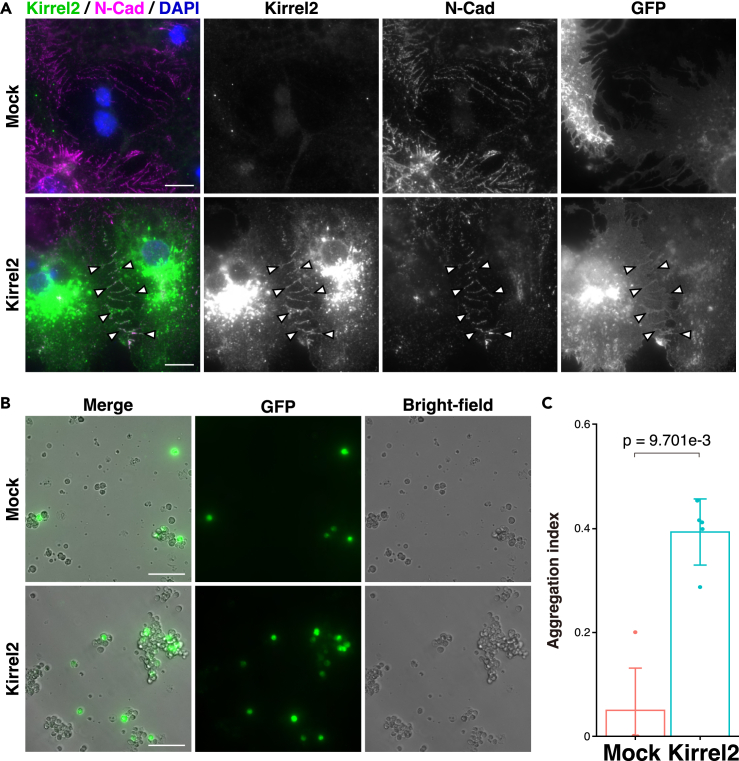
Localization of Kirrel2 in cultured astrocytes (A) Immunofluorescence images of astrocytes transfected with Kirrel2 and GFP-mem immunostained with the indicated Abs. N-Cad, N-cadherin. Arrowheads, boundary between neighboring cultured astrocytes. Scale bars, 10 μm. (B and C) Cell aggregation assay of astrocytes transfected with Kirrel2 and GFP-mem. Scale bars, 100 μm. The aggregation index was calculated by dividing the number of GFP-positive aggregated cells by the total number of GFP-positive cells. Data are presented as mean and SD (n = 5), and dot shows each data. The Mann-Whitney U test was performed. These images are representative of three independent experiments. See also Figure S13.

Lastly, we evaluated the cell-cell adhesion activity of Kirrel2 in astrocytes because it has not been reported that Kirrel2 has cell adhesion activity in astrocytes. Primary astrocytes were prepared from dissociated neurospheres stimulated with 1% FBS.^21^^,^^41^ More than 95% of the cells expressed astrocyte markers, such as AQP4 and EAAT1 (Figure S13A), consistent with previous reports.^21^^,^^41^^,^^42^ As primary astrocytes formed the intricate structure of terminal processes, and endogenous expression levels of Kirrel2 were low, both membrane-anchored GAP-43 fused to GFP (GFP-mem) and Kirrel2 proteins were overexpressed in primary astrocytes (Figure S13B). The intense Kirrel2 signal was observed at the boundary between neighboring astrocytes (Figure 12A). The Kirrel2 signal co-localized with another CAM N-cadherin signal (Figure 12A). We next examined the cell adhesion activity of Kirrel2 using a cell aggregation assay. Aggregates of Kirrel2- and GFP-mem-transfected primary astrocytes were larger than those of control cells (Figures 12B and 12C). Collectively, these results indicate that Kirrel2 potentially has cell adhesion activity of cultured astrocytes at least *in vitro*.

## Discussion

In this study, we biochemically isolated brain blood vessels from mouse brain homogenates and isolated PV-AEF from the isolated blood vessels. Electron microscopic analysis revealed that the purified PV-AEF consisted of various sizes of cell fragments and vesicles. We performed mass spectrometry analysis on the purified PV-AEF and identified 9,762 proteins in the purified PV-AEF, in which mitochondria-related proteins were enriched, compared with the isolated blood vessels, whereas extracellular matrix proteins and transcription-related proteins were not. We focused here on three CAMs, nectin-2δ,^21^ Kirrel2/Neph3/filtrin,^25^^,^^26^^,^^27^ and podoplanin/aggrus/gp36/E11.^28^^,^^29^^,^^30^^,^^31^ We identified Kirrel2 and podoplanin as novel PV-AEF molecules. Nectin-2δ and podoplanin localized mainly in PV-AEF of arteries/arterioles and veins/venules, but hardly in those of capillaries, whereas Kirrel2 localized mainly in PV-AEF of arteries/arterioles, but hardly in those of capillaries or veins/venules. We also found that nectin-2α/δ, Kirrel2, and podoplanin were preferentially observed in large sizes of PV-AEF. These results revealed that PV-AEF had various sizes and different molecular components, implying different roles of PV-AEF in NVU depending on different vascular regions.

Recently, another isolation method of PV-AEF from that shown in this study was reported and identified 516 proteins, including numerous critical electron transport chain proteins and BBB-related proteins.^36^ In this method, brain blood vessels were dissociated into a single-cell suspension by treatment with 100 μg/mL Liberase DL and 20 U/mL DNase I for 60 min, and PV-AEF were isolated using magnetic activation cell sorting (MACS) system with an anti-astrocyte cell surface antigen-2 antibody (Ab). Proteolytic digestion at these high concentrations and long incubation periods for PV-AEF dissociation might affect the degradation of cell surface proteins, and it is not well understood whether astrocyte cell surface antigen-2 is expressed in all PV-AEF, although the MACS system is a valuable way to isolate PV-AEF. To improve these two weaknesses of this method as much as possible, we dissociated PV-AEF from the isolated blood vessels with Liberase DL and DNase I at low concentrations (37.5 μg/mL Liberase DL and 2 U/mL DNase I) for 15 min incubation and isolated PV-AEF biochemically using Ficoll density gradient centrifugation. The present method is helpful for the isolation of various types and sizes of PV-AEF. Indeed, the known components of PV-AEF, such as AQP4, dystroglycan, syntrophin, dystrophin, dystrobrevin, glucose transporter 1, TRPV4, integrin α6, sideroflexin-5,^36^ MLC1,^16^^,^^17^^,^^18^ and GlialCAM,^19^ were included in common 9,353 proteins. The purified PV-AEF contained different sizes of cell fragments and vesicles, consistent with the different sizes of PV-AEF on brain blood vessels *in situ* and *in vivo*.

Brain blood vessels are mostly wrapped with PV-AEF.^2^^,^^18^ Consistently, the AQP4 astrocyte marker signal was observed in almost all the laminin α4-positive blood vessels *in vivo*. It was recently shown that mature oligodendrocytes in gray matter directly associate with the vascular BM.^43^ In this report, ∼17% of oligodendrocytes mainly contact with capillaries, but not with arterioles or venules, in the mouse cerebral cortex, hippocampus, and cerebellum. In this study, we did not observe the MBP oligodendrocyte marker signal on the isolated blood vessels, but our mass spectrometry analysis showed that oligodendrocyte marker proteins, such as Mag, Mog, and Cntn2, were detected in the purified PV-AEF fraction. Although the source of these oligodendrocyte proteins identified in the purified PV-AEF fraction was unclear, they may be derived from blood-vessel-associated oligodendrocytes. Further investigations are needed for making this conclusion.

Diphtheria-toxin-receptor-mediated ablation targeted to *Mlc1*- or *Slc1a3*-expressing astrocytes induces plasma protein leakage and downregulation of CAMs in VECs, leading to BBB breakdown.^44^^,^^45^ Astrocytes are thought to act as a link between blood vessels and neurons in the brain by regulating BBB permeability, blood flow, metabolite availability, and neuronal activity.^2^^,^^4^^,^^5^^,^^6^^,^^8^ Single astrocyte contacts at least one blood vessel, and most single astrocytes contact three blood vessels in the somatosensory cortex.^46^ In addition, some astrocytes are tightly associated with blood vessels through their somata, called juxtavascular astrocytes.^47^ However, the molecular components and morphology of PV-AEF in different vascular regions and brain regions have not been well understood. Recent vascular single-cell RNA transcriptome analysis revealed that the transcriptional profiles of VECs and mural cells are different depending on vascular regions in mice,^22^ raising the possibility that the expression profiles of PV-AEF have regional differences depending on vascular regions. Consistently, we observed the different regional localization of nectin-2δ, Kirrel2, and podoplanin in mouse brain blood vessels. In addition, we observed the different sizes of PV-AEF on brain blood vessels *in situ* and *in vivo*, and the sizes of PV-AEF in large blood vessels seem to be larger than those in small blood vessels. To support this observation, the expression levels of Kirrel2, which were observed in arteries/arterioles, and those of nectin-2δ and podoplanin, which were observed in arteries/arterioles and veins/venules, were higher in large sizes of the purified PV-AEF. These present results are in good agreement with the earlier observations in the mouse brain that the sizes of PV-AEF increase with vessel diameter and that this size variation is most pronounced in arteries/arterioles rather than veins/venules, although the heterogeneity of PV-AEF in molecular components was not shown.^18^ In this previous report, the sizes of PV-AEF in the mouse brain were analyzed *in vivo* using an anti-MLC1 Ab, because MLC1 localizes at the boundary between neighboring PV-AEF in mouse, rat, and human brain tissues.^17^^,^^18^ The role of this size variation of PV-AEF remains unclear, but computational modeling simulations suggest that it is involved in relatively constant flux between perivascular and interstitial compartments.^18^ It was previously shown that proteins are synthesized in PV-AEF, sustaining their structural and functional polarization.^34^ Therefore, it could be speculated that each endfoot process of highly ramified astrocytes has specific functions and morphology depending on vascular regions. In addition, different brain regions have different vascular permeability and disease susceptibility in the mouse brain,^48^ suggesting that there may be differences in PV-AEF depending on brain regions. Thus, the isolation of PV-AEF or astrocytes by MACS system with an anti-nectin-2δ, anti-Kirrel2, or anti-podoplanin Ab and comparison of the expression profiles might provide further insight into the different roles of PV-AEF and heterogeneity of astrocytes. The precise molecular components of PV-AEF are essential for our understanding of the regulatory mechanisms of BBB in NVU by astrocytes. Furthermore, protein expression profiles of transporters and receptors in brain microvessels in human are different from those in mice,^49^ indicating that BBB properties are different among species.^49^^,^^50^ Comparison of PV-AEF components across species is necessary for elucidating the species differences of BBB.

Mutations of PV-AEF molecules, such as MLC1 and GlialCAM, cause a human developmental disease, called megalencephalic leukoencephalopathy with subcortical cyst, in which gliovascular functions are dysregulated.^51^^,^^52^ Thus, the integrity of BBB depends on both cell-cell adhesion and cell-matrix adhesion.^3^^,^^4^^,^^5^^,^^6^ We focused here on three CAMs, nectin-2δ, Kirrel2, and podoplanin, among identified proteins in the purified PV-AEF. Expression of these three CAMs is also enriched in perivascular astrocytes.^24^ Nectin-2δ, also known as CD112 or PVR-related 2 (PVRL2/PRR2), is a Ca^2+^-independent CAM.^20^ We previously showed that nectin-2δ localizes at the boundary between PV-AEF and BM of blood vessels in the mouse brain and that genetic ablation of *nectin-2* causes degeneration of PV-AEF.^21^ At one month of age of *nectin-2*-deficient mice, the electron density of the cytoplasm in PV-AEF is increased, and the plasma membrane of PV-AEF protrudes toward the abluminal side. At six months of age, *nectin-2*-deficient mice show the atrophy of white and gray matters and the enlargement of the lateral ventricles, although the detailed molecular mechanisms of these phenotypes remain unclear. The present study revealed that nectin-2δ localized mainly in PV-AEF of arteries/arterioles and veins/venules. Because BM components differ depending on vascular regions,^22^^,^^53^ these different molecular components of BM may regulate the localization of nectin-2δ. Genome-wide association study revealed that the human *NECTIN-2* gene is associated with Alzheimer's disease.^20^^,^^54^ Comparison of PV-AEF components between *nectin-2*-deficient and wild-type mice may provide insight into the mechanisms for the degeneration of PV-AEF and pathological aspect of Alzheimer's disease.

Kirrel2 is one of the Kirrel family members with Ig-like domains.^25^^,^^26^^,^^27^ The Kirrel superfamily members show homophilic cell adhesion activity, which is necessary for their functions.^37^^,^^38^^,^^39^ In the mouse olfactory bulb and accessory olfactory bulb, homophilic adhesion of Kirrel2 is essential for axonal segregation into distinct glomeruli.^37^^,^^38^^,^^39^ In the developing mouse cerebellum and spinal cord, Kirrel2 is expressed in early postmitotic neural precursors and GABAergic progenitor-selective cells and regulates neuronal differentiation.^55^^,^^56^ However, the role of Kirrel2 in astrocytes remains unclear. We showed here that Kirrel2 localized mainly in PV-AEF of arteries/arterioles, but hardly in those of capillaries or veins/venules, in the mouse brain, and that Kirrel2 potentially had cell adhesion activity of cultured astrocytes at least *in vitro*. Because electron microscopic 3D reconstitution analysis using the rat brain revealed that PV-AEF cover most of the surface of brain blood vessels and attach to each other,^35^ Kirrel2 may localize at the boundary between neighboring PV-AEF of arteries/arterioles and play a role in adhesion between neighboring PV-AEF *in vivo*. Consistently, Kirrel2 is also expressed in the glomerular podocytes, which cover the glomerular capillaries in the kidney.^57^ Kirrel2 localizes at the boundary between neighboring podocyte foot processes, called slit diaphragms, which contribute to the glomerular filtration barrier.^57^ Considering these previous observations, it could be speculated that Kirrel2 localizing at the boundary between neighboring PV-AEF of arteries/arterioles may contribute to the regulation of the BBB permeability and influx and efflux transport. Of note, in cerebral amyloid angiopathy, deposition of amyloid, such as amyloid β protein, is observed in the tunica media and adventitia of the arteries and arterioles in the human cerebral cortex, which may lead to neurodegenerative diseases through vascular dysfunction.^58^^,^^59^ It would be interesting if Kirrel2 localizing at the boundary between neighboring PV-AEF of arteries/arterioles is involved in cerebral amyloid angiopathy. Further studies are needed to clarify the role of Kirrel2 at the boundary between neighboring PV-AEF of arteries/arterioles.

Podoplanin is a mucin-type glycoprotein and has multiple physiological functions associated with lymphangiogenesis, platelet aggregation, immune response, and oncogenesis.^31^ Podoplanin contributes to the formation of membrane-actin structures through the interaction with ezrin/radixin/moesin proteins to activate RhoA signaling.^31^^,^^60^ Interestingly, podoplanin plays an important role in maintaining the normal morphology of podocyte foot processes and glomerular permeability in the kidney.^31^^,^^61^ In puromycin aminonucleoside nephrosis, a rat model of human minimal change nephropathy, the extensive flattening of podocyte foot processes and severe proteinuria are observed in association with the high reduction of podoplanin expression.^28^ Treatment of anti-podoplanin neutralization Ab in rat leads to proteinuria in association with extensive flattening of foot processes.^61^ It could be speculated that podoplanin localizing in PV-AEF of arteries/arterioles and veins/venules regulates the different morphology of PV-AEF, which controls the BBB function in NVU, although we did not reveal here whether it localized at the boundary between PV-AEF and BM, between neighboring PV-AEF, or both, or at the intracellular organellar membrane of PV-AEF. Recently, podoplanin expression correlates with microglial activation, including cell mobility and phagocytosis, after traumatic brain injury in a mouse model,^62^ raising the possibility that upregulation of podoplanin by some pathological conditions may affect astrocyte morphology and mobility, leading to the induction of activated astrocytes. Because half of *Pdpn*-deficient mice die in the first week,^31^ a conditional knockout technology is useful for clarifying the role of podoplanin in the morphology of PV-AEF and regulation of BBB.

### Limitations of the study

In this study, because we dissociated PV-AEF from the isolated blood vessels with Liberase DL and DNase I, proteolytic digestion might affect the degradation of PV-AEF components. Because we isolated PV-AEF biochemically using Ficoll density gradient centrifugation, cell fragments and vesicles derived from blood-vessel-associated cells, including fibroblasts, oligodendrocytes, oligodendrocyte precursor cells, and microglia, could not be completely separated from PV-AEF. The heterogeneity of PV-AEF in different brain regions remains to be pursued. We analyzed the PV-AEF components of C57BL/6J mouse, but not other species. Comparative studies on PV-AEF components among various species should be performed in future because BBB properties are different among species. It also remains unclear whether the heterogeneity of PV-AEF is observed in other species, including humans. In this study, we showed that Kirrel2 potentially had cell adhesion activity of cultured astrocytes at least *in vitro*. However, the direct evidence for the adhesion between neighboring PV-AEF *in vivo* remains to be determined. We attempted to perform the cell aggregation assay using the purified PV-AEF, but the purified PV-AEF did not aggregate with each other in our experimental conditions, although they may contain many CAMs. The exact reason for the inability of the purified PV-AEF to aggregate with each other is not known but may be because the adhesion activity of many CAMs other than cadherins is very weak. Furthermore, the function and molecular mechanisms of the identified molecules enriched in PV-AEF remain to be pursued, because it is crucially important for our understanding of the regulatory mechanisms of BBB in NVU and their disorders. The graphical abstract shows that nectin-2δ localizes at the boundary between PV-AEF and BM of arteries/arterioles and veins/venules based on our present and previous findings,^21^ that Kirrel2 tentatively localizes at the boundary between neighboring PV-AEF of arteries/arterioles, and that podoplanin tentatively localizes at the boundary between PV-AEF and BM of arteries/arterioles and veins/venules, because the detailed localizations of Kirrel2 and podoplanin remain unclear.

## STAR★Methods

### Key resources table

**Table:** 

REAGENT or RESOURCE	SOURCE	IDENTIFIER
**Antibodies**		

alpha-Tubulin Antibody	Cell Signaling Technology	Cat# 2144, RRID:AB_2210548
Anti-Actin Antibody, clone C4	Millipore	Cat# MAB1501, RRID:AB_2223041
Anti-AQP4 antibody produced in rabbit	Atlas Antibodies	Cat# HPA014784, RRID:AB_1844967
Mouse Anti-Actin, alpha-Smooth Muscle Monoclonal Antibody, Unconjugated, Clone 1A4	Sigma-Aldrich	Cat# A5228, RRID:AB_262054
CD 31 Platelet Endothelial Cell Adhesion Molecule (PECAM)	BD Biosciences	Cat# 550274, RRID:AB_393571
Recombinant Anti-CD31 Antibody [EPR17260-263]	Abcam	Cat# ab222783, RRID:AB_2905525
Mouse/Rat CD31/PECAM-1 Antibody	R and D Systems	Cat# AF3628, RRID:AB_2161028
Cleaved Caspase-3 (Asp175) (5A1E) Rabbit mAb	Cell Signaling Technology	Cat# 9664, RRID:AB_2070042
Collagen IV Monoclonal Antibody (PHM-12, CIV22)	Thermo Fisher Scientific	Cat# MA5-14100, RRID:AB_10982391
Anti-GFAP Antibody	Abcam	Cat# ab134436, RRID:AB_2818977
Anti-GFP	Nacalai Tesque	Cat# 04404-84, RRID:AB_10013361
GLAST (Plasmalemmal Glutamate Transporter GLAST) Antibody	Frontier Institute	Cat# GLAST-GP, RRID:AB_2571717
Anti Iba1, Rabbit Antibody	Fujifilm-Wako	Cat# 019-19741, RRID:AB_839504
Goat Anti-Mouse Kirrel2 Polyclonal Antibody, Unconjugated	R and D Systems	Cat# AF2930, RRID:AB_2265276
Lamin B1 Antibody	Proteintech	Cat# 12987-1-AP, RRID:AB_2136290
Anti-Laminin Antibody Produced in Rabbit	Sigma-Aldrich	Cat# L9393, RRID:AB_477163
Monoclonal Anti-Laminin-2 (alpha-2 Chain) Antibody Produced in Rat	Sigma-Aldrich	Cat# L0663, RRID:AB_477153
Goat Anti-Mouse Laminin alpha 4 Affinity Purified Polyclonal Antibody, Unconjugated	R and D Systems	Cat# AF3837, RRID:AB_2249744
Myelin Basic Protein Antibody	Novus	Cat# NB600-717, RRID:AB_2139899
MLC1 (N50) Antibody	Wang et al.^18^	N/A
N-Cadherin (D4R1H) XP Rabbit Antibody	Cell Signaling Technology	Cat# 13116, RRID:AB_2687616
Rat Anti-Mouse Nectin 2 Monoclonal Antibody, Unconjugated, Clone 502-57	MBL International	Cat# D083-3, RRID:AB_590848
Recombinant Anti-Nectin 2 Antibody [EPR6717]	Abcam	Cat# ab135246, RRID:AB_2936435
Anti-NG2 Chondroitin Sulfate Proteoglycan	Millipore	Cat# AB5320, RRID:AB_91789
Mouse PDGF R alpha Antibody	R and D Systems	Cat# AF1062, RRID:AB_2236897
PDGFR-beta (958)	Santa Cruz Biotechnology	Cat# sc-432, RRID:AB_631068
Recombinant Anti-Podoplanin / gp36 Antibody [PMab-1]	Abcam	Cat# ab256559, RRID:AB_2936436
VDAC1/2 Antibody	Proteintech	Cat# 10866-1-AP, RRID:AB_2257153
Von Willebrand Factor Antibody	Novus	Cat# NB600-586, RRID:AB_10002749
Anti-IgG (H+L chain) (Goat) pAb-HRP	MBL International	Cat# 546, RRID:AB_2936437
Sheep Anti-Mouse IgG - Horseradish Peroxidase	GE Healthcare	Cat# NA931, RRID:AB_772210
Goat Anti-Rabbit IgG - H&L Polyclonal Antibody, Hrp Conjugated	Abcam	Cat# ab6721, RRID:AB_955447
Cy3-AffiniPure Donkey Anti-Chicken IgY (IgG) (H+L) (min X Bov,Gt,GP,Sy Hms,Hrs,Hu,Ms,Rb,Rat,Shp Sr Prot)	Jackson ImmunoResearch Labs	Cat# 703-165-155, RRID:AB_2340363
Donkey anti-Goat IgG (H+L) Cross-Adsorbed Secondary Antibody, Alexa Fluor™ 488	Thermo Fisher Scientific	Cat# A-11055, RRID:AB_2534102
Donkey anti-Goat IgG (H+L) Cross-Adsorbed Secondary Antibody, Alexa Fluor™ 555	Thermo Fisher Scientific	Cat# A-21432, RRID:AB_2535853
Donkey anti-Goat IgG (H+L) Cross-Adsorbed Secondary Antibody, Alexa Fluor™ 647	Thermo Fisher Scientific	Cat# A-21447, RRID:AB_2535864
Brilliant Violet 421-AffiniPure Donkey Anti-Guinea Pig IgG (H+L) (min X Bov,Ck,Gt,Sy Hms,Hrs,Hu,Ms,Rb,Rat,Shp Sr Prot)	Jackson ImmunoResearch Labs	Cat# 706-675-148, RRID:AB_2651104
Anti-Guinea Pig IgG (H+L), Highly Cross-adsorbed, CF™555 Antibody Produced in Donkey	Sigma-Aldrich	Cat# SAB4600297, RRID:AB_2814810
Cy3-AffiniPure Donkey Anti-Mouse IgG (H+L) (min X Bov,Ck,Gt,GP,Sy Hms,Hrs,Hu,Rb,Rat,Shp Sr Prot)	Jackson ImmunoResearch Labs	Cat# 715-165-151, RRID:AB_2315777
Donkey Anti-Mouse IgG H&L (Alexa Fluor® 750) Preadsorbed	Abcam	Cat# ab175739, RRID:AB_2936438
Donkey anti-Rabbit IgG (H+L) Highly Cross-Adsorbed Secondary Antibody, Alexa Fluor™ 488	Thermo Fisher Scientific	Cat# A-21206, RRID:AB_2535792
Cy5-AffiniPure Donkey Anti-Rabbit IgG (H+L) (min X Bov,Ck,Gt,GP,Sy Hms,Hrs,Hu,Ms,Rat,Shp Sr Prot)	Jackson ImmunoResearch Labs	Cat# 711-175-152, RRID:AB_2340607
Donkey Anti-Rat IgG H&L (Alexa Fluor® 488) Preadsorbed	Abcam	Cat# ab150153, RRID:AB_2737355
Donkey Anti-Rat IgG H&L (Alexa Fluor® 555) Preadsorbed	Abcam	Cat# ab150154, RRID:AB_2813834
NOT AN ANTIBODY Streptavidin, Alexa Fluor® 555 Conjugate Antibody	Thermo Fisher Scientific	Cat# s21381, RRID:AB_2307336

**Chemicals, peptides, and recombinant proteins**		

HBSS(+) with Ca, Mg, without Phenol Red, Liquid	Nacalai Tesque	Cat# 09735-75
1 mol/l-HEPES Buffer Solution	Nacalai Tesque	Cat# 17557-94
Fetal Bovine Serum (FBS)	Sigma-Aldrich	Cat# 173012
Ficoll PM400	Cytiva	Cat# 17030050
cOmplete™, Protease Inhibitor Cocktail	Roche	Cat# 11836170001
Immobilon ECL Ultra Western HRP Substrate	Merck	Cat# WBULS0100
PARAFORMALDEHYDE EXTRA PURE DAC	Merck	Cat# 1.04005.1000
P-APMSF, HYDROCHLORIDE	Nacalai Tesque	Cat# 178281
Sucrose	Fujifilm-Wako	Cat# 193-07921
Sodium Pyruvate (100 mM)	Thermo Fisher Scientific	Cat# 11360070
PBS Tablets (Phosphate Buffered Salts)	Takara	Cat# T900
Tissue Tek O.C.T. Compound	Sakura Finetek	Cat# 4583
HistoVT One (10x, pH 7.0)	Nacalai Tesque	Cat# 06380-05
FluorSave Reagent	Merck	Cat# 345789
10% Glutaraldehyde Solution	Fujifilm-Wako	Cat# 071-02031
Epok812	Okenshoji	Cat# 02-1001
DNase I	Sigma-Aldrich	Cat# D4263-1VL
Liberase DL	Roche	Cat# 5401160001
Albumin, from Bovine Serum (BSA), pH5.2 (Fraction V)	Fujifilm-Wako	Cat# 013-21275
Normal Goat Serum	Abcam	Cat# ab7481
Donkey Serum	Millipore	Cat# S30-100ML
4',6-Diamidino-2-phenylindole Dihydrochloride (DAPI)	Nacalai Tesque	Cat# WBULS0100
Lipofectamine 2000	Thermo Fisher Scientific	Cat# 11668019

**Critical commercial assays**		

Pierce BCA Protein Assay Kit	Thermo Fisher Scientific	Cat# 23227
CellStain Double Staining Kit	Dojindo	Cat# 341-07381
DeadEnd Fluoremetric TUNEL System	Promega	Cat# G3250

**Deposited data**		

Raw and Analyzed Mass Spectrometry Data	This paper	PXD037949 / JPST001913

**Experimental models: Organisms/strains**		

C57BL/6JJcl	Japan CLEA	RRID:IMSR_JCL:MIN-0003

**Recombinant DNA**		

pMX-Neph3 Vector	Minaki et al.^55^	N/A
pBI-CMV1 Vector	Takara	Cat# 631630
pAcGFP1-mem Vector	Takara	Cat# 632468
pBI-GFPmem Vector	This study	N/A
pBI-GFPmem-Kirrel2 Vector	This study	N/A

**Software and algorithms**		

Adobe Photoshop	Adobe	https://www.adobe.com RRID:SCR_014199
Adobe Illustrator	Adobe	https://www.adobe.com RRID:SCR_010279
Fiji / ImageJ	Schindelin et al.^63^	https://imagej.net/software/fiji/ RRID:SCR_002285
Perseus	Max Planck institute of biochemistry	https://maxquant.net/perseus/ RRID:SCR_015753
R package	R core team	https://www.r-project.org RRID:SCR_003005
RStudio	Posit	https://posit.co RRID:SCR_000432

**Other**		

100 μm Cell Strainer	BD Falcon	Cat# 352360
40 μm Cell Strainer	BD Falcon	Cat# 352340
pluriStrainer 20 μm	pluriSelect	Cat# 43-50020-03

### Resource availability

#### Lead contact

Further information and requests for resources and reagents should be directed to and will be fulfilled by the lead contact, Yoshimi Takai (ytakai@med.kobe-u.ac.jp).

#### Materials availability

Materials are available upon request.

### Experimental model and study participant details

#### Animals

Male C57BL/6J mice purchased from CLEA were maintained under a 12/12 light-dark cycle. All animal experiments were conducted in accordance with the regulations for animal experimentation of Kobe University. This study was approved by the president of Kobe University after being reviewed by the Animal Care and Use Committee of Kobe University (approval number: 30-04-03).

#### Primary astrocytes

Mouse astrocyte cultures were prepared by differentiation of neurospheres derived from the mouse ganglionic eminence of male or female brain at embryonic day 18.5 as described previously.^21^^,^^41^ For differentiation of astrocytes, neurospheres were treated with 1% FBS.

### Method details

#### Isolation of mouse brain blood vessels

Biochemical isolation of brain blood vessels from eight-week-old male mice was performed using a slightly modified protocol based on previously published studies.^32^^,^^33^ In brief, the brains of three mice were homogenized on ice using a Potter-Elvehjem homogenizer (0.25 mm clearance, 400 rpm, 20 strokes) in 10 ml H+H buffer (Hanks’ balanced salt solution (+) with Ca^2+^, Mg^2+^ (Nacalai Tesque) containing 10 mM HEPES (Nacalai Tesque)) with 4% FBS (Sigma-Aldrich) (S0 fraction). The homogenate was mixed thoroughly with 15 ml of 30% Ficoll PM400 (GE Healthcare) and centrifuged at 4,500 × *g* for 15 min at 4°C (himac CP80α, P28S swing rotor, Hitachi). The pellet was suspended in 3 ml of H+H buffer with 1% FBS (S1 fraction) and passed through a 100- or 40-μm nylon mesh filter (Falcon). The mesh filters were gently washed in H+H buffer to obtain brain blood vessels (S3 and S5 fractions, respectively). Each fraction was passed through a 20-μm mesh filter (pluriSelect) and gently washed to remove contaminated cells from brain blood vessels.

#### Western blotting

Western blotting was performed as described previously.^21^ In brief, mouse brain homogenate and the isolated blood vessels were lysed with RIPA buffer (50 mM Tris-HCl, pH 7.4, 1% NP-40, 0.5% sodium deoxycholate, 0.1% SDS, 150 mM NaCl) supplemented with a protease inhibitor cocktail (Roche). The cell lysates were incubated on ice for 15 min and centrifuged at 12,000 × *g* at 4°C for 15 min. The protein concentration was determined by BCA protein assay (Pierce). The supernatant was mixed with 5 x Laemmli sample buffer and boiled at 95°C for 5 min. The samples were subjected to SDS-PAGE and transferred to polyvinylidene difluoride membranes. The membranes were blocked with 5% BSA, and then incubated with primary Abs, followed by incubation with horseradish peroxidase (HRP)-conjugated secondary Abs. The signals were visualized by incubation with Immobilon Western Chemiluminescent HRP Substrate (Merck Millipore), and then detected using the ImageQuant LAS4000 (GE Healthcare). Abs used in this study were shown in Table S4.

#### Cell viability assay of the isolated brain blood vessels

For cell viability assay, the isolated brain blood vessels were stained using CellStain Double Staining Kit (Dojindo) according to the manufacturer’s protocol. For TUNEL staining, DeadEnd Fluorometric TUNEL System (Promega) was used according to the manufacturer’s protocol.

#### Immunofluorescence microscopic analysis

Immunofluorescence microscopic analysis was performed as described previously.^21^^,^^65^ In brief, the isolated blood vessels were spun onto glass slides at 800 × *g* for 3 min using Cytospin 4 (Thermo Fisher Scientific). Samples were fixed with 2% paraformaldehyde at 37°C for 15 min and incubated with 0.25% Triton X-100 in PBS(-) at room temperature for 10 min. The samples were incubated with 1% BSA in PBS(-) at room temperature for 30 min and subsequently incubated with 10% donkey or goat serum at room temperature for 30 min. The samples were immunostained with the primary Abs at room temperature overnight, washed and then immunostained with appropriate fluorophore-conjugated secondary Abs (1:300). For brain tissue immunostaining, deeply anesthetized mice were transcardially perfused with 0.4 M phosphate buffer (PB, pH7.4) supplemented with heparin and (p-amidinophenyl)methanesulfonyl fluoride, followed by perfusion with 2% paraformaldehyde, 4% sucrose, 1 mM sodium pyruvate, Hanks’ balanced salt solution containing 1 mM CaCl_2_ and 1 mM MgCl_2_. Dissected brain was incubated in the same fixative at 4°C for 4 h and then dehydrated in 100 mM PB containing 20% sucrose (pH7.4) for 2 h, followed by incubation in 100 mM PB containing 25% sucrose (pH7.4) overnight. The brains were placed in Tissue Tek O.C.T. Compound (Sakura Finetek) and frozen. The sections of 12-μm thickness were mounted on glass slides and incubated in HistoVT One (Nacalai Tesque), at 62°C for 20 min, and then with 10% donkey serum at room temperature for 1 h. The specimens were incubated with primary Abs at 4°C overnight, washed and then immunostained with secondary Abs and 1 μg/ml DAPI (Nacalai Tesque) in the PBS(-) at room temperature for 2 h. The samples were mounted in FluorSave reagent (Merck Millipore). Images were captured with a confocal laser-scanning microscope (LSM 510 META, CarlZeiss) or a fluorescence microscope (BZ-X710, Keyence). Abs used in this study were shown in Table S4. For biotinylation of anti-VWF Ab, Ab-10 Rapid Biotin Labeling Kit (Dojindo) was used according to the manufacturer’s protocol. The sizes of PV-AEF and the fluorescence intensity were measured by Fiji software (version 2.3.0, ImageJ2).^63^ For quantification of the ratio of PV-AEF protein signal-positive blood vessels, multiple hippocampus and cerebral cortex images were obtained and the number of brain blood vessels was counted manually, and their percentages were calculated.

#### Electron microscopic analysis

The specimens were fixed with 2.5% glutaraldehyde in 0.1 M PB (pH 7.4), followed by post fixation with 2% OsO_4_ in the same buffer. For SEM analysis, the specimens were dehydrated with a graded series of ethanol and subjected to critical point drying using a CPD300 (Leica). These specimens were mounted on aluminum stubs with carbon paste, these specimens were coated with osmium with a Neoc-Pro osmium coater (Meiwafosis) and observed with a field-emission scanning electron microscope (S-4800, Hitachi). Cell fragments and vesicles attached to the isolated blood vessels were colorized manually using Adobe Photoshop CC software. The area and length of PV-AEF were measured by Fiji software (version 2.3.0, ImageJ2).^63^ For TEM analysis, the specimens were dehydrated with a graded series of ethanol and embedded in Epok812 (Okenshoji). Ultrathin sections were cut and stained with uranyl acetate and lead citrate. These sections were examined with a transmission electron microscope JEM-1400 Flash (JEOL).

#### Isolation of the purified PV-AEF

The isolated brain blood vessels (defined as B0 fraction) were treated with 2 U/ml DNase I (Sigma-Aldrich) and 37.5 μg/ml Liberase DL (Roche) in H+H buffer at 37°C for 15 min. The sample was pipetted gently and immediately passed through a 20-μm mesh filter and gently washed in H+H buffer to obtain PV-AEF-detached blood vessels (B1 fraction). The flow-through sample was centrifuged at 3,000 × *g* for 5 min, and the pellet was suspended in 1 ml of H+H buffer (crude PV-AEF). For preparation of the discontinuous Ficoll density gradient, Ficoll PM400 [30% (w/v, density = 1.11 g/ml) and 10% (w/v, density = 1.03 g/ml)] was made from the bottom to the top of a 15 ml Falcon tube. Ficoll gradient was made by layering 5 ml of each Ficoll solution. One ml of the crude PV-AEF was added on the top of the gradient and centrifuged at 800 × *g* for 60 min. After centrifugation, 11 fractions were collected and diluted in five times the volume of PBS(-). Each fraction was centrifuged at 20,000 × *g* for 30 min and washed in PBS(-). Fractions 6–11 (density, < 1.04 g/ml) were used as the purified PV-AEF.

#### Mass spectrometry of the purified PV-AEF and data analysis

B0 fraction, the crude PV-AEF, and the purified PV-AEF were prepared from eight-week-old male mice (n = 12). Four independent experiments were performed for replicates. Cell pellets were solubilized with phase transfer surfactant buffer^66^ and boiled at 95°C for 5 min. Then, 50 μg of B0 fraction and all amount of the crude PV-AEF and the purified PV-AEF were reduced with 10 mM Tris(2-carboxyethyl)phosphine, alkylated with 20 mM iodoacetamide, and protein was purified using the single-pot, solid-phase-enhanced sample-preparation (SP3) protocol.^67^ Purified protein was digested with trypsin and Lys-C for 16 h at 37°C. Peptides were desalted and purified on a C18-SCX StageTips.^68^ Peptides were dried up with Speedvac and solubilized with 0.1% formic acid/2% acetonitrile.

LC-MS/MS was performed by coupling an UltiMate 3000 Nano LC system (Thermo Scientific) and an HTC-PAL autosampler (CTC Analytics) to an Orbitrap Fusion Lumos mass spectrometer (Thermo Fisher Scientific). Peptides were delivered to an analytical column (75 μm × 30 cm, packed in-house with ReproSil-Pur C18-AQ, 1.9 μm resin, Dr. Maisch, Ammerbuch, Germany) and separated at a flow rate of 280 nL/min using a 105-min gradient from 5% to 30% of solvent B (solvent A, 0.1% formic acid; solvent B, 0.1% formic acid and 99.9% acetonitrile). The Orbitrap Fusion Lumos instrument was operated in the data-independent acquisition mode (DIA). For gas-phase fractionation-DIA acquisition of a pooled B0 fraction for library, full mass spectra were acquired with the following parameters: a resolution of 120,000, an automatic gain control (AGC) target of 4 × 10^6^ with a maximum injection time of 250 ms. The five gas-phase fractionation-DIA runs collectively covered 398–802 *m/z* (i.e., 398–482, 478–562, 558–642, 638–722, and 718–802 *m/z*). MS2 spectra were collected with the following parameters: a 2-*m/z* isolation window at 50,000 resolution, an AGC target of 4 × 10^5^ ions, a maximum injection time is 86 ms, and normalized collision energy of 30. For the individual samples for proteome profiling acquisition, full mass spectra were acquired in the range of 400–800 *m/z* with the following parameters: a resolution of 120,000, an AGC target of 4 × 10^5^ with a maximum injection time of 100 ms. MS2 spectra were collected with the following parameters: an 8-*m/z* isolation window at 50,000 resolution, an AGC target of 4 × 10^5^ ions, a maximum injection time is 86 ms, overlapping window patterns and normalized collision energy of 30.

Raw MS data were processed with DIA-NN software (version 1.7.12).^69^ Database searching included all entries from the *Mus musculus* UniProt database and contaminant database.^70^ The search parameters were as follows: up to one missed cleavage sites, 7–30 peptide length, carbamidomethylation of cysteine residues as static modifications, protein names from FASTA for implicit protein grouping, robust LC (high precision) for quantification strategy, and RT-dependent for cross-run normalization. Precursor ions were adjusted to a 1% false discovery rate (FDR). MaxLFQ was used for label-free quantifications.^71^ Perseus (version 2.0.7.0) was used for statistical analysis, filtering by GO term.^72^ For visualizing data, the statistical package R was used for UMAP (“umap” and “ggplot2” package) and heatmap and hierarchical clustering analysis (“pheatmap” package, complete linkage method). GO terms that are enriched in each cluster were identified from GO Biological Processes 2021 using Enrichr (https://maayanlab.cloud/Enrichr/) with the default parameters. NaN values in replicates were deleted from the calculation of the average.

#### Plasmid construction

The cDNA for mouse *Kirrel2* was obtained from pMX-Neph3 (gift from Yuichi Ono, KAN Research Institute Inc., Japan)^55^ and cloned into pBI-CMV1 vector (Takara). For membrane anchored-GFP, GFP sequence of pBI-CMV1 was replaced by GFP-mem sequence of pAcGFP1-mem (Takara) (pBI-GFPmem).

#### Astrocyte culture and transfection

For differentiation of astrocytes, neurospheres were dissociated by pipetting and treated with 1% FBS. For transfection, Lipofectamine 2000 (Thermo Fisher Scientific) was used according to the manufacturer’s protocol. One day after transfection, transfected cells were seeded on poly-L-lysine coated coverslip in 24-well plate and incubated for four days. The transfection efficiency of astrocytes was approximately 30%. All cells were maintained in culture at 37°C in a humidified 5% CO_2_ incubator.

#### Cell aggregation assay

Six days after transfection, cells were washed with PBS(-) and detached from the dishes using 0.01% trypsin. Detached cells were harvested, resuspended in H+H buffer and pipetted into single cells. 2 × 10^5^ cells were seeded onto 6-well ultra-low attachment plates. The plates were then placed on a nutator at 37°C for 45 min and imaged using a fluorescence microscope (BZ-X710, Keyence). The aggregation index was calculated by dividing the number of GFP-positive aggregated cells by the total number of GFP-positive cells.

#### *In silico* data collection and analysis

Physiological expression of *Kirrel2* and *Pdpn* in the mouse brain was analyzed and created using the online single cell RNA-seq database and tools [Allen Brain Map, whole cortex & hippocampus – 10X genomics (2020) with 10X-smart-seq taxonomy (2021), https://portal.brain-map.org/atlases-and-data/rnaseq; Betsholz dataset,^22^^,^^73^ http://betsholtzlab.org/VascularSingleCells/database.html]. *Kirrel2* and *Pdpn* expression in astrocytes of mouse visual cortex during mouse development was analyzed by the online database (GSE161398, http://igc1.salk.edu:3838/astrocyte_transcriptome/).^74^ The expression profile of perivascular astrocytes and non-perivascular astrocytes and its comparison was created from McCarty dataset (“EnhanacedVolcano” package).^24^

### Quantification and statistical analysis

All experiments were repeated at least three times. Statistical analysis for the differential expression was performed with ANOVA test with permutation-based FDR (FDR < 0.05) using Perseus software (version 2.0.7.0). Statistical analysis of the difference between mean values was performed with the Mann-Whitney U test using R software (version 3.6.2). The ratio of PV-AEF protein signal-positive blood vessels in each vascular regions was counted manually and created bar graphs. The statistical significance was set at p < 0.05.

## Data Availability

• All data reported in this paper is available from the lead contact upon request. The mass spectrometry raw data and processed data are deposited to the ProteomeXchange Consortium via the jPOST repository with the dataset identifier PXD037949 / JPST001913,^64^ which are publicly accessible. • This paper does not report the original code. • Any additional information required to reanalyze the data reported in this paper is available from the lead contact upon request.

## Supplemental information

Document S1. Figures S1–S13

Table S1. Mass spectrometry analysis of all identified proteins, related to Figures 4 and S5

Table S2. Protein lists and its average expression, related to the Venn diagrams of Figure 4A

Table S3. Significant differential expression proteins, related to Figures 4B and 5

Table S4. Abs used in this study, related to STAR★METHODS
