# Supplementary material for: Heterogeneity of perivascular astrocyte endfeet depending on vascular regions in the mouse brain
Source: iScience. 2023 Sep 21;26(10):108010. doi: 10.1016/j.isci.2023.108010 (PMC10565786; doi:10.1016/j.isci.2023.108010)
Supplement: Document S1. Figures S1–S13 [file mmc1.pdf]

iScience, Volume *26*

Supplemental information

Heterogeneity of perivascular astrocyte endfeet depending on vascular regions in the mouse brain

Takeshi Kameyama, Muneaki Miyata, Hajime Shiotani, Jun Adachi, Soichiro Kakuta, Yasuo Uchiyama, Kiyohito Mizutani, and Yoshimi Takai

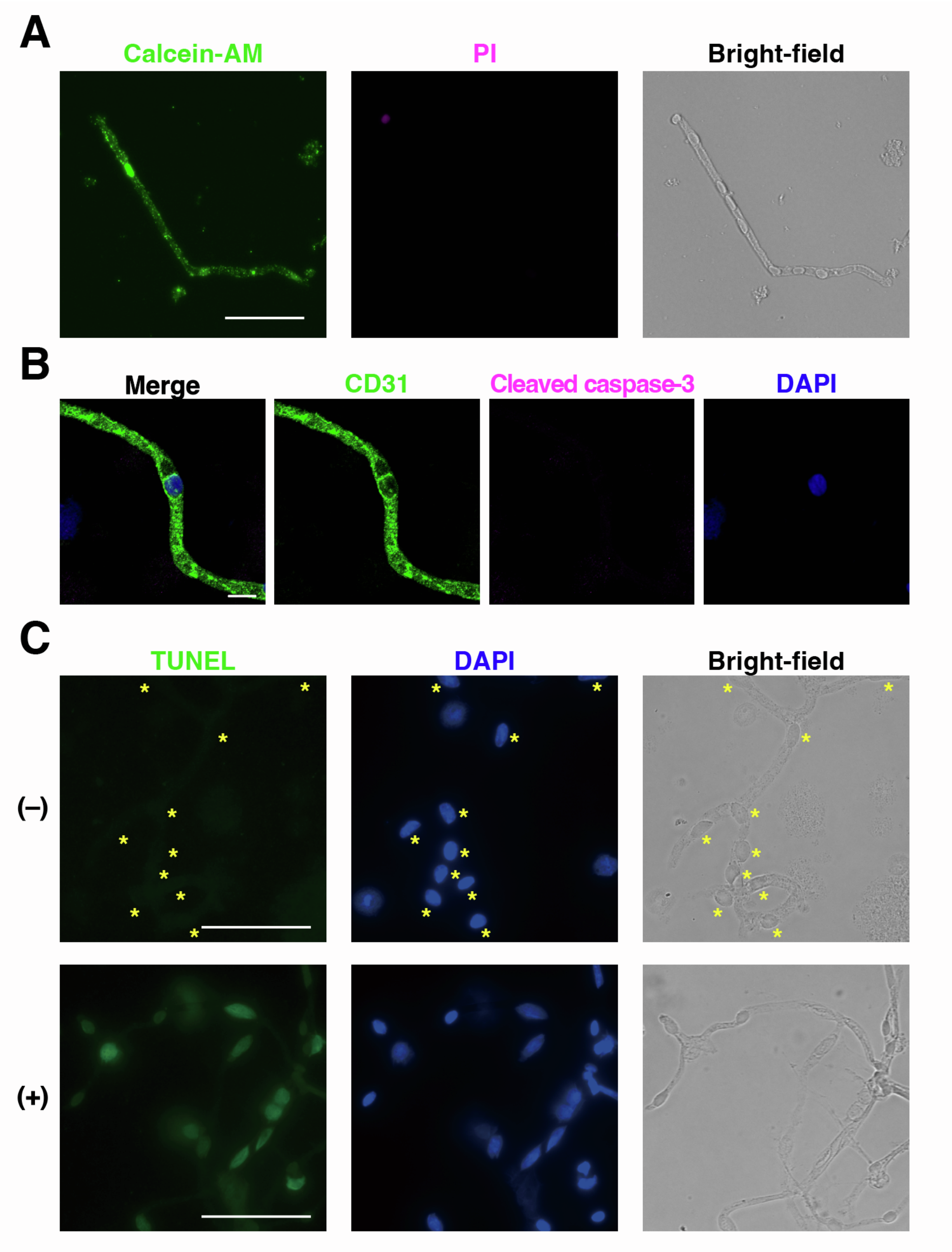

Figure S1. Viability of vascular cells in the isolated blood vessels, related to Figure 1.
(A) The isolated blood vessels (mixture of S3 and S5 fractions) were stained with calcein-AM and propidium iodide (PI) to analyze the viability of vascular cells. Scale bar, 50 μm. (B) Immunofluorescence images of the isolated mouse brain vessels immunostained with the indicated Abs. Scale bar, 10 μm. (C) TUNEL and DAPI staining of the isolated blood vessels (–). (+) indicates DNase I treatment, for positive control of TUNEL staining. *, TUNEL-negative cells in the isolated blood vessels. Scale bars, 50 μm. Representative capillaries of the isolated blood vessels are shown. These images are representative of three independent experiments.

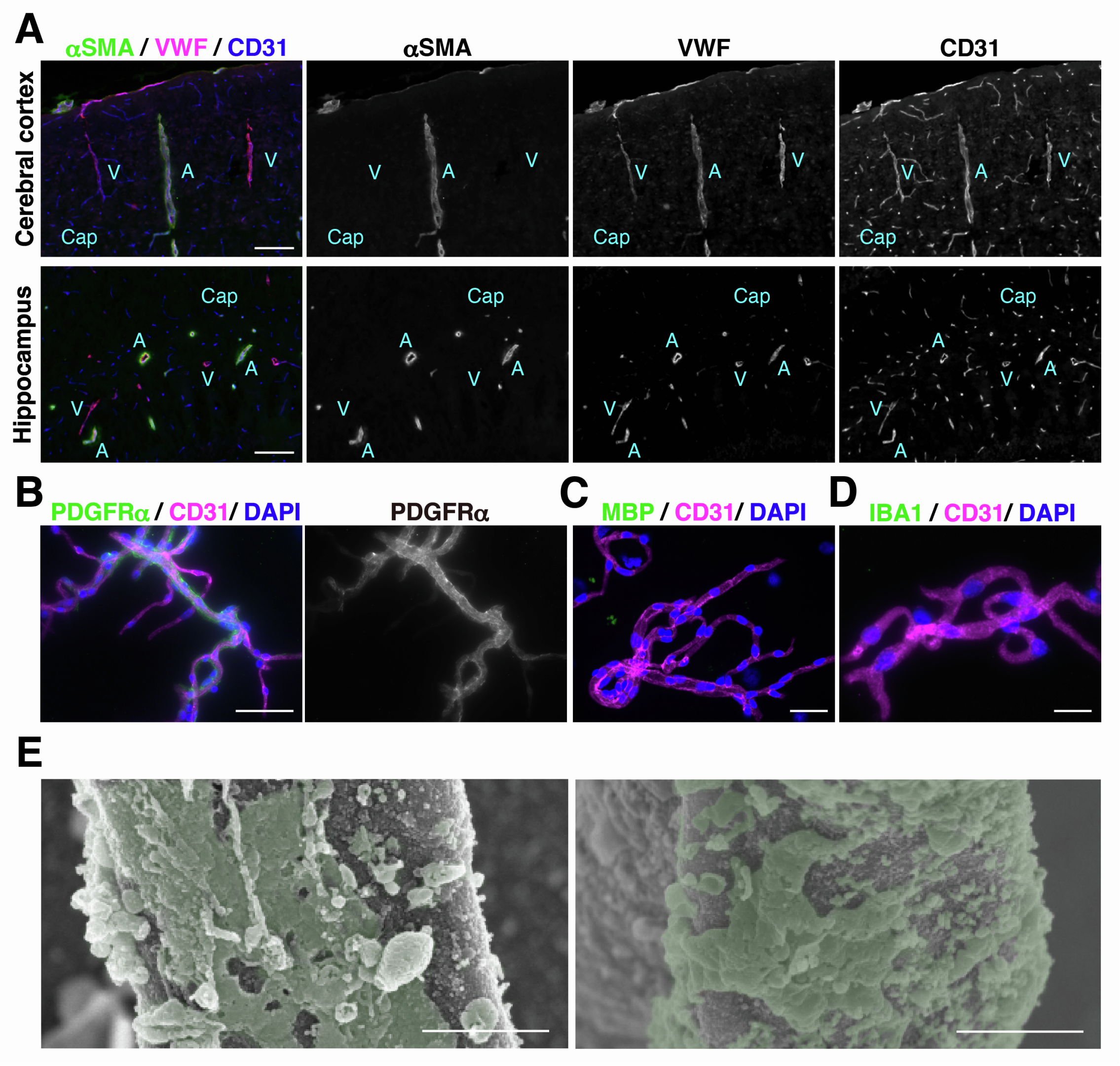

Figure S2. Identification of arteries/arterioles, capillaries, and veins/venules in the mouse cerebral cortex and hippocampus, related to Figure 2.

Immunofluorescence images of the eight-week-old male mouse brain immunostained with the indicated Abs. Top, cerebral cortex. Bottom, hippocampus. A, artery/arteriole; V, vein/venule; and Cap, capillary. Scale bars, 100 μm. (B–D) Immunofluorescence images of the isolated blood vessels (mixture of S3 and S5 fractions) immunostained with the indicated Abs. Scale bars, 50 μm. (E) SEM analysis of the isolated blood vessels. Cell fragments and vesicles were colored in green. Original images were shown in Figure 2C. Scale bars, 1 μm. These images are representative of three independent experiments.

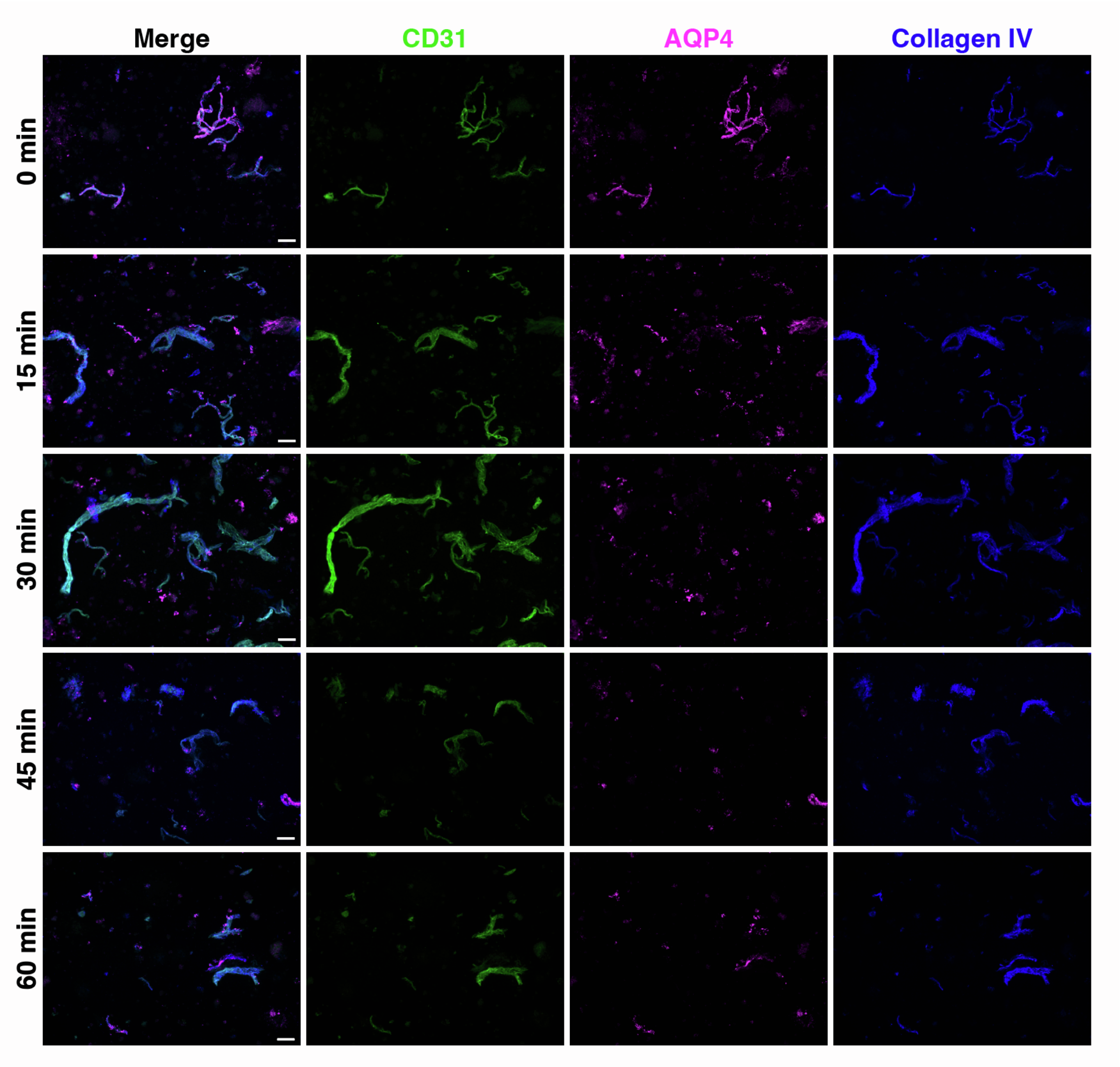

Figure S3. Detachment of PV-AEF from the isolated blood vessels by enzyme treatment, related to Figure 3.

The isolated mouse brain vessels treated with Liberase DL and DNase I for the indicated time were immunostained with the indicated Abs. Scale bars, 100 μm. These images are representative of three independent experiments.

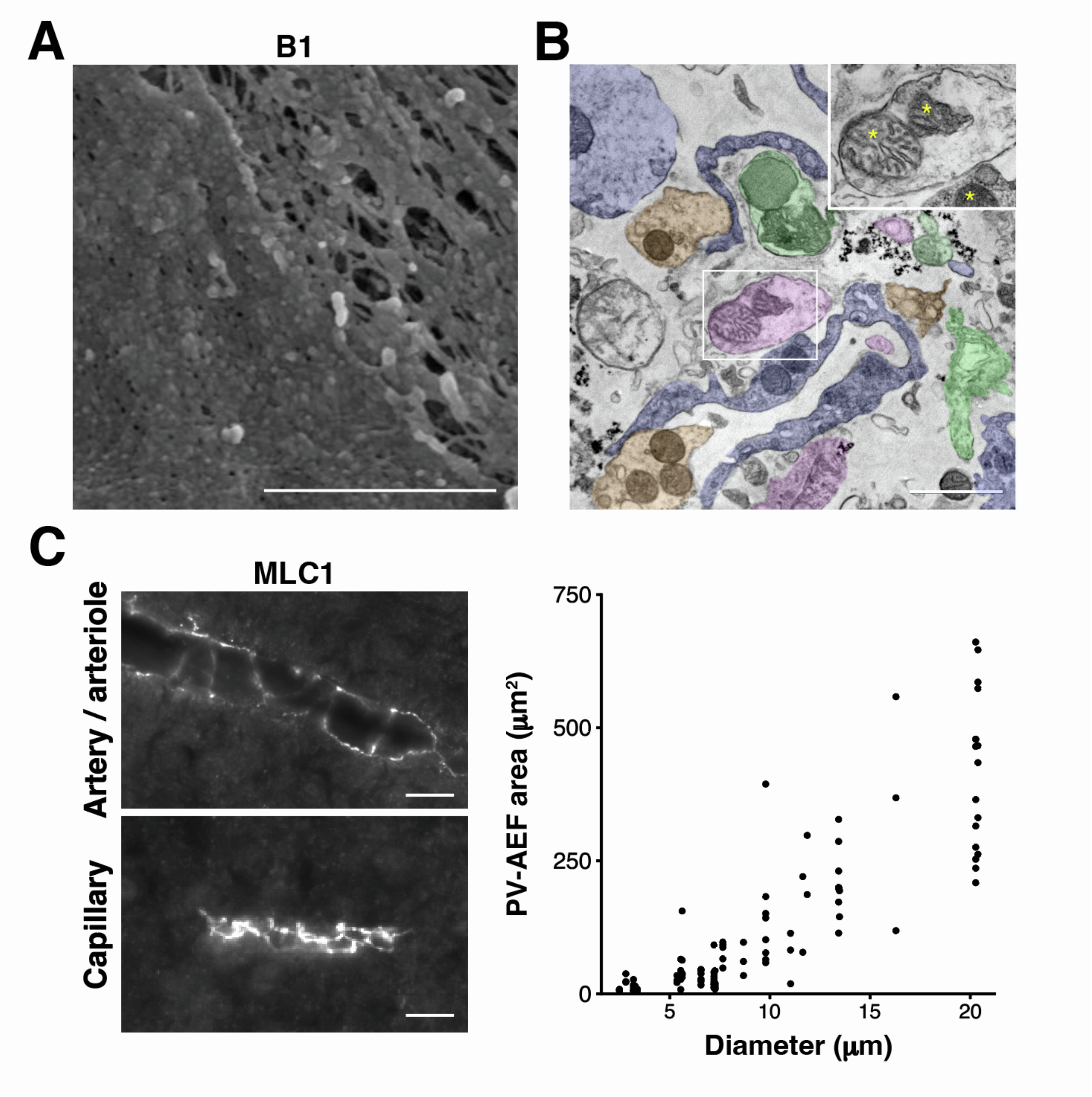

Figure S4. Structure of PV-AEF-detached blood vessels and the sizes of PV-AEF, related to Figure 3.

(A) SEM analysis of B1 fraction. Scale bar, 1 μm. (B) TEM analysis of the purified PV-AEF. Each of putative PV-AEF is shown in each color. Boxed region is magnified in inset. *, mitochondria. Original images are shown in Figure 3G. Scale bar, 1 μm. (C) Immunofluorescence images of the eight-week-old male mouse cerebral cortex immunostained with MLC1 Ab. Scale bars, 20 μm. Individual PV-AEF areas were plotted against vessel diameters (right).

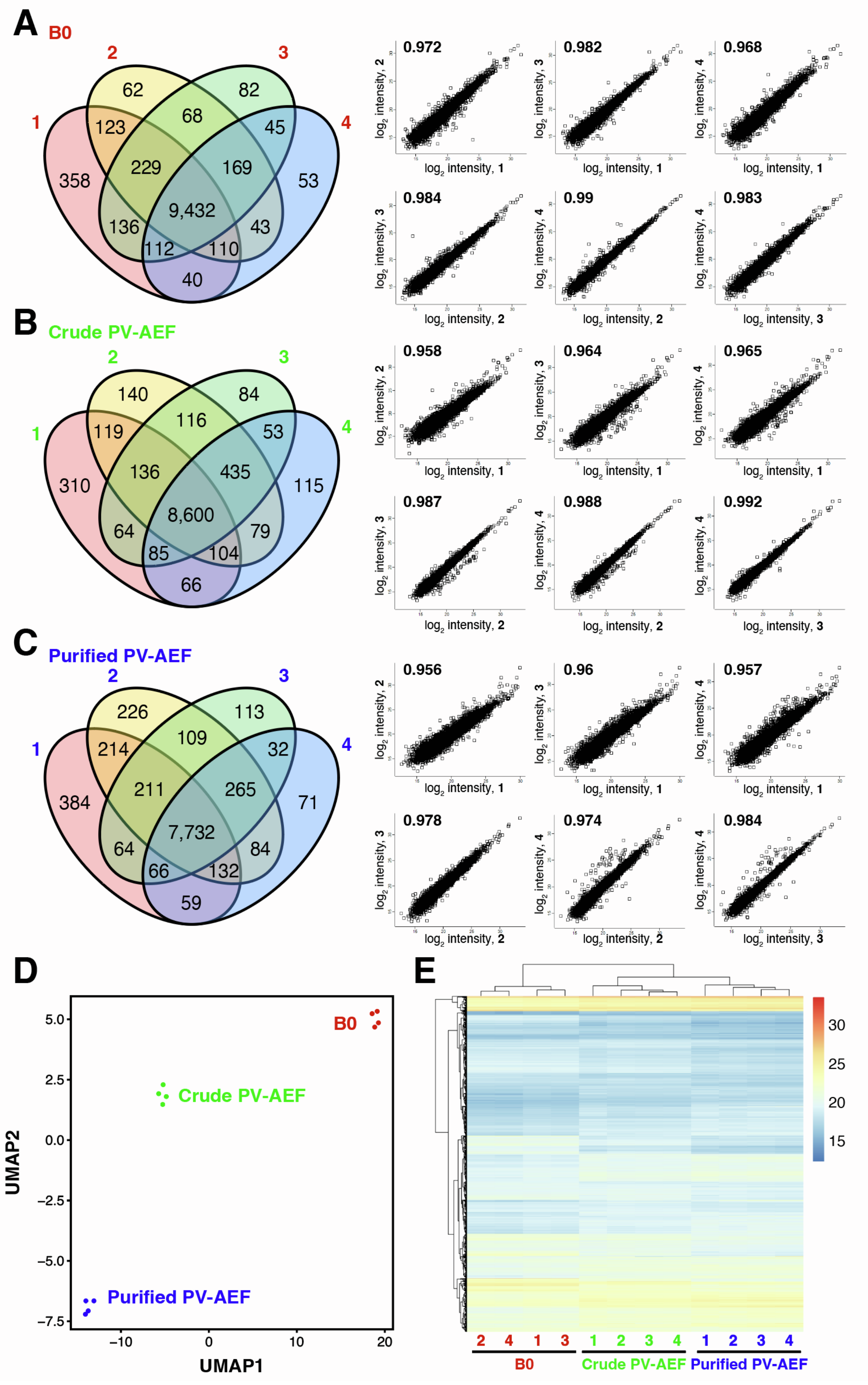
A
B0
1
2
3
4
358
62
82
53
123
68
45
229
169
9,432
136
43
112
110
40
0.972
0.982
0.968
0.984
0.99
0.983
log2 intensity, 1
log2 intensity, 2
log2 intensity, 3
log2 intensity, 4
B
Crude PV-AEF
310
140
84
115
119
116
53
136
435
8,600
64
79
85
104
66
0.958
0.964
0.965
0.987
0.988
0.992
C
Purified PV-AEF
384
226
113
71
214
109
32
211
265
7,732
64
84
66
132
59
0.956
0.96
0.957
0.978
0.974
0.984
D
B0
Crude PV-AEF
Purified PV-AEF
UMAP1
UMAP2
5.0
2.5
0.0
−2.5
−5.0
−7.5
−10
0
10
20
E
30
25
20
15
2 4 1 3
1 2 3 4
1 2 3 4
B0
Crude PV-AEF
Purified PV-AEF

Figure S5. Mass spectrometry analysis of each sample, related to Figure 4 and Table S1.

(A–C) Venn diagrams with the number of proteins common to or distinct from each other in four biological replicates (No. 1–4) of B0 (A), the crude PV-AEF (B), and the purified PV-AEF (C) (left). Scatter plots of the log_2 intensity of identified proteins between the indicated replicates, and its Pearson correlation shows strong similarities of each sample (right). B0, the mixture of S3 and S5 fractions. (D) UMAP representation of B0 (red dots), the crude PV-AEF (green dots), and the purified PV-AEF (blue dots). (E) Heatmap and hierarchical clustering of all identified proteins. The color scale reflects log_2 intensity of each sample. The complete linkage method was used for hierarchical clustering. These analyses reveal that higher similarity of each sample.

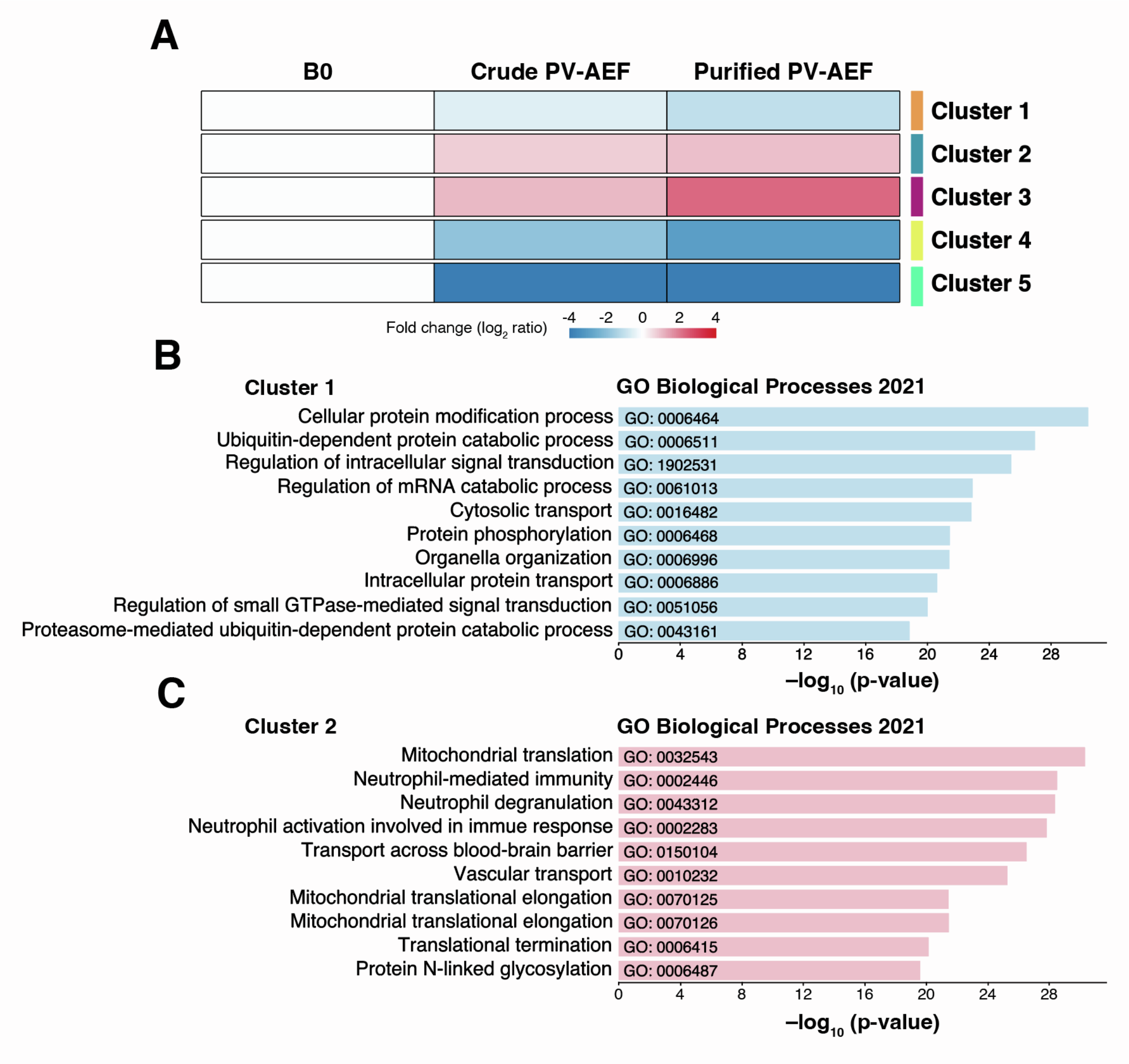

Figure S6. Mass spectrometry analysis of the purified PV-AEF, related to Figure 4 and Tables S2 and S3.

(A) Heatmap shows average $\log_2$-fold changes of all proteins in the indicated clusters related to Figure 4B. (B and C) Gene ontology enrichment analysis of biological processes for the indicated clusters related to Figure 4B. The top 10 enriched GO Biological Processes 2021 are shown.

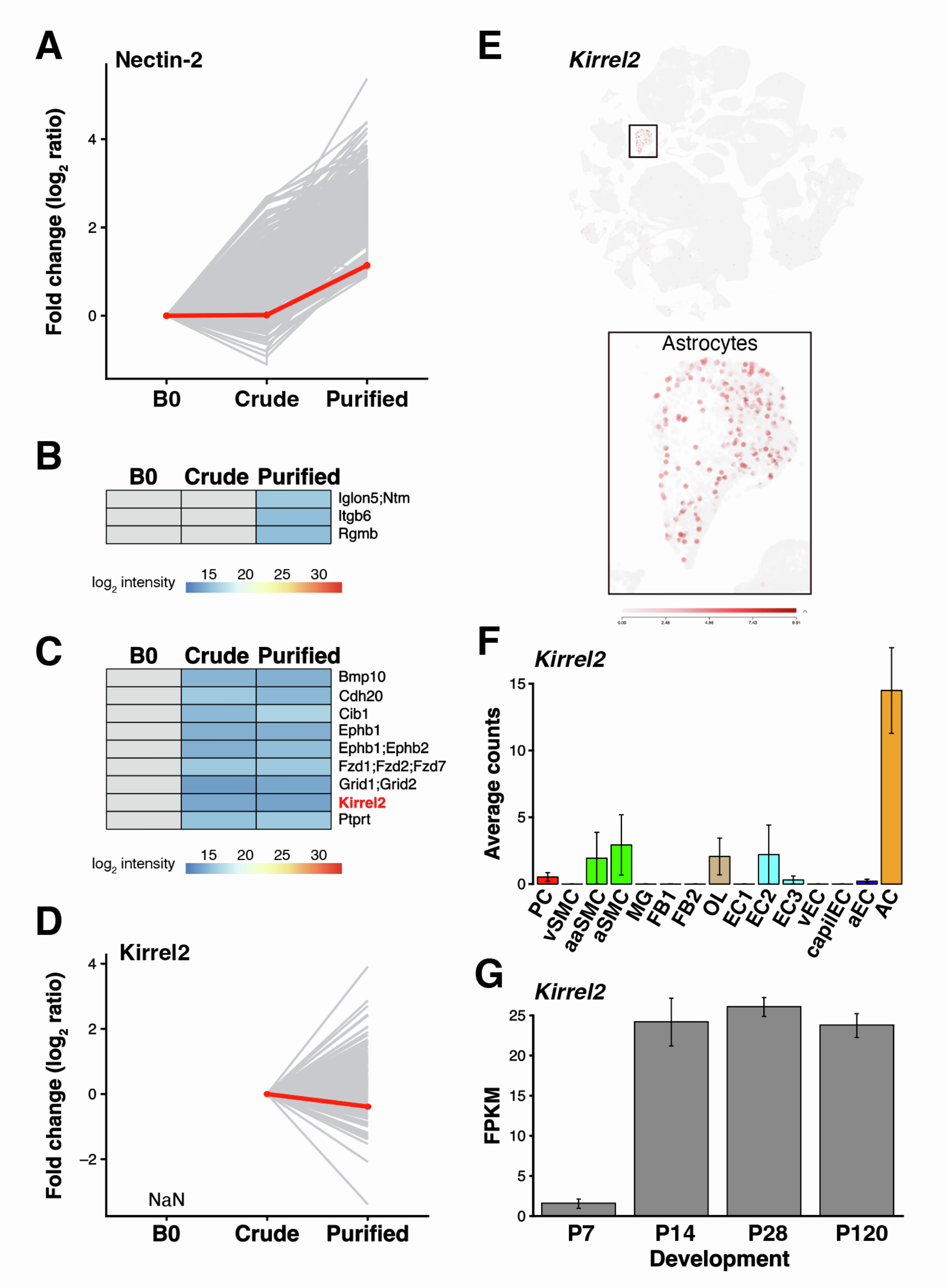

Figure S7. Mass spectrometry analysis of CAMs and *in silico* RNA sequencing analysis of *Kirrel2* expression in the mouse brain, related to Figure 4 and Table S3.

(A) Average log_2-fold changes of individual Cluster 3 proteins are shown. Red line, nectin-2; gray lines, other proteins in Cluster 3. (B) Heatmap shows average log_2 intensity of the indicated cell adhesion proteins that were identified in 83 proteins specific for the purified PV-AEF (n = 4). Gray, NaN value. (C) Heatmap shows average log_2 intensity of the indicated cell adhesion proteins that were commonly observed in the purified PV-AEF and the crude PV-AEF (n = 4). Gray, NaN value.

(D) Average log_2-fold changes of individual 188 proteins that were commonly observed in the purified PV-AEF and the crude PV-AEF are shown. Red line, Kirrel2; gray lines, other proteins in the 188 proteins. (E) UMAP projection of *Kirrel2* expression from single cell RNA-sequencing dataset of Allen Brain Map. (F) *Kirrel2* expression from single cell RNA-sequencing dataset. [S1, S2] Bar plot of the average expression levels of *Kirrel2* in each cluster. Cell type annotations for each cluster are indicated. PC, pericytes; SMC, smooth muscle cells; MG, microglia; FB, vascular fibroblast-like cells; OL, oligodendrocytes; EC, endothelial cells; AC, astrocytes; v, venous; c, capillary; a, arterial; aa, arteriolar; and 1,2,3, subtypes. (G) *Kirrel2* expression in astrocytes of mouse visual cortex at the indicated developmental timepoints from GFAP-Ribotag mice. [S3] FPKM, fragments per kilobase of exon per million reads mapped.

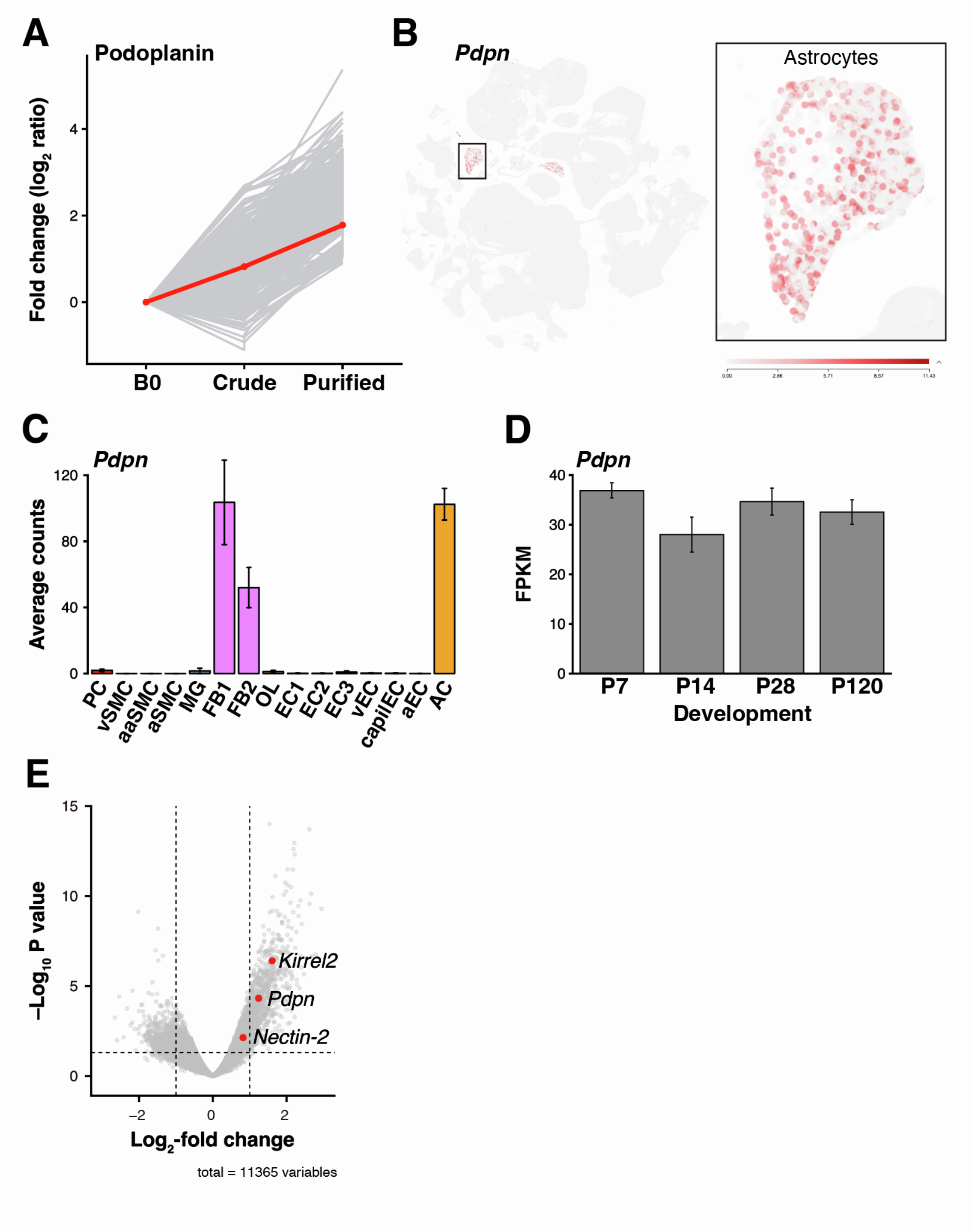

Figure S8. Mass spectrometry analysis of CAMs and *in silico* RNA sequencing analysis of *Pdpn* expression in the mouse brain, related to Figure 5 and Table S3.

(A) Average $\log_2$-fold changes of individual Cluster 3 proteins are shown. Red line, podoplanin; gray lines, other proteins in Cluster 3. (B) UMAP projection of *Pdpn* expression from single cell RNA-sequencing dataset of Allen Brain Map. (C) *Pdpn* expression from single cell RNA-sequencing dataset. [S1, S2] Bar plot of the average expression levels of *Pdpn* in each cluster.

Cell type annotations for each cluster are indicated. PC, pericytes; SMC, smooth muscle cells; MG, microglia; FB, vascular fibroblast-like cells; OL, oligodendrocytes; EC, endothelial cells; AC, astrocytes; v, venous; c, capillary; a, arterial; aa, arteriolar; and 1,2,3, subtypes. (D) *Pdpn* expression in astrocytes of mouse visual cortex at the indicated developmental timepoints from GFAP-Ribotag mice. [S3] FPKM, fragments per kilobase of exon per million reads mapped. (E) Volcano plot for the comparison between perivascular astrocytes vs non-perivascular astrocytes from McCarty dataset. [S4] Significantly differentially expressed genes are shown in each dot. *Nectin-2*, *Kirrel2, and Pdpn* are shown in red dots. The horizontal dashed line indicates the adjusted p-value = 0.01 and the vertical dashed lines indicate $\log_2$-fold changes = ±1.

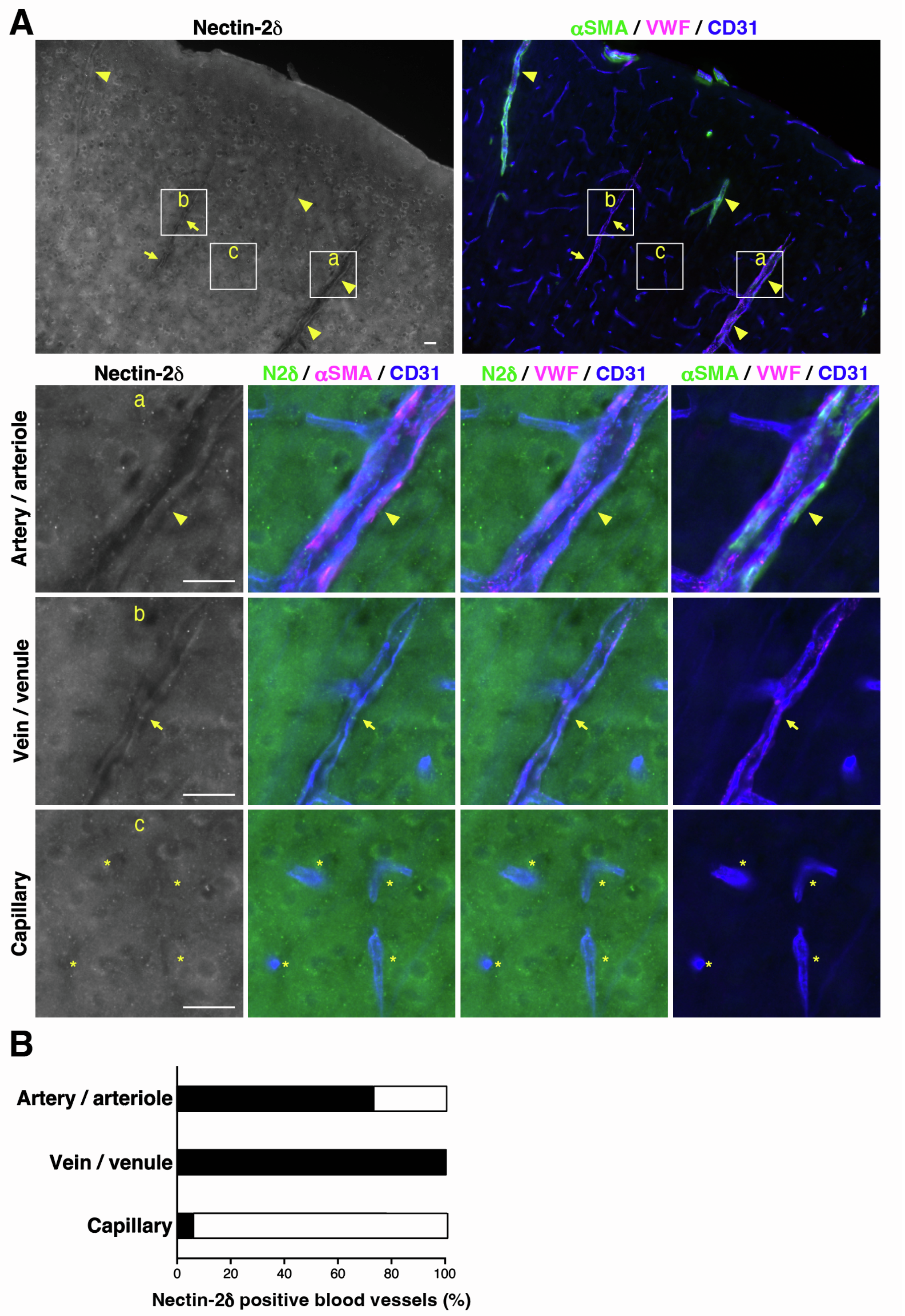
A
Nectin-2δ
αSMA / VWF / CD31
b
c
a
Nectin-2δ
N2δ / αSMA / CD31
N2δ / VWF / CD31
αSMA / VWF / CD31
Artery / arteriole
Vein / venule
Capillary
B
Artery / arteriole
Vein / venule
Capillary
0
20
40
60
80
100
Nectin-2δ positive blood vessels (%)

Figure S9. Localization of nectin-2δ in the mouse cerebral cortex, related to Figure 6.

(A) Immunofluorescence images of the eight-week-old male mouse cerebral cortex immunostained with the indicated Abs. N2δ, nectin-2δ. a, artery/arteriole; b, vein/venule; and c, capillaries. Arrowheads, arteries/arterioles; Arrows, veins/venules; *, capillaries. Scale bars, 20 μm. (B) The ratio of nectin-2δ-positive blood vessels in the mouse cerebral cortex is shown as a bar graph. These images are representative of three independent experiments.

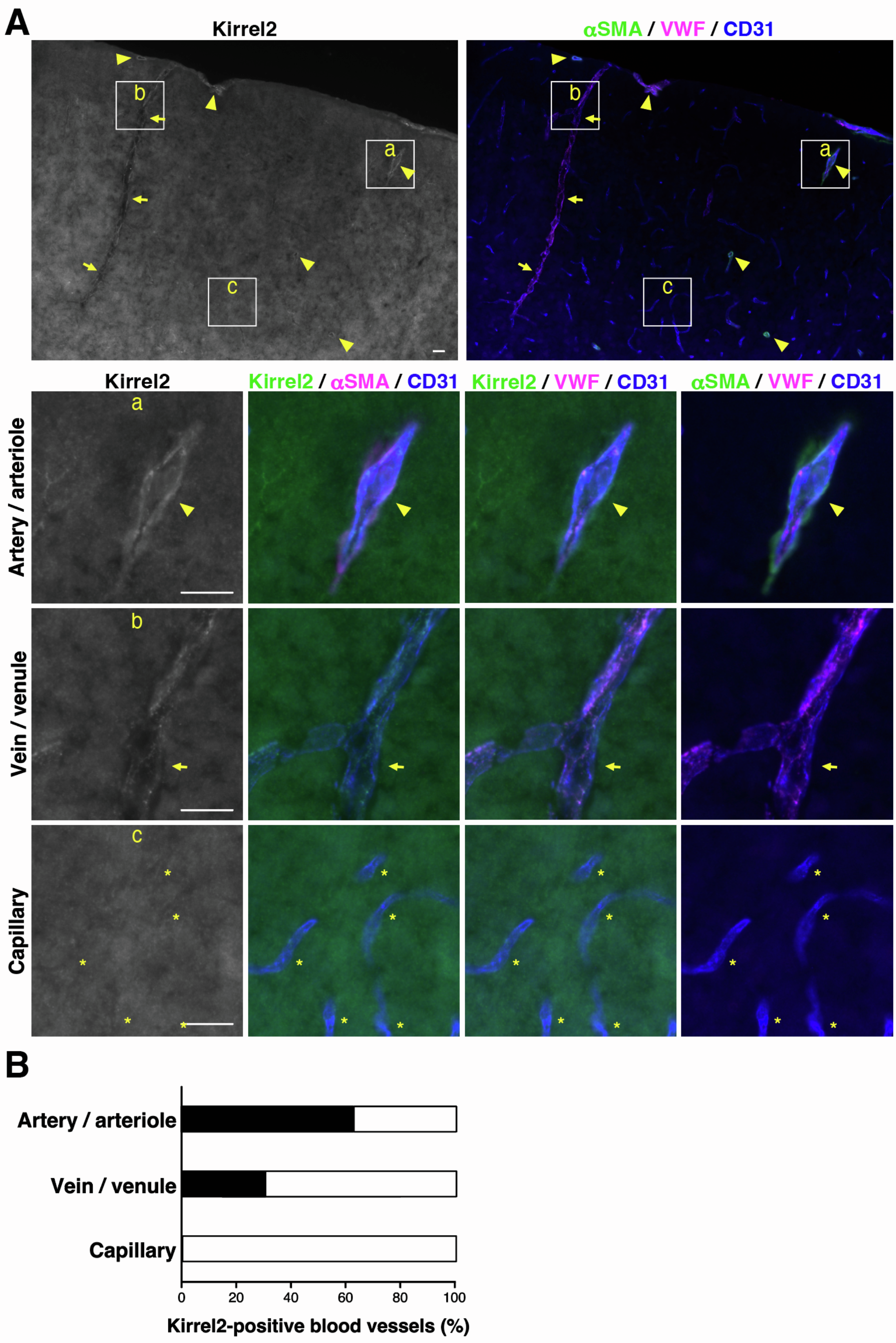
A
Kirrel2
αSMA / VWF / CD31
a
b
c
Kirrel2
Kirrel2 / αSMA / CD31
Kirrel2 / VWF / CD31
αSMA / VWF / CD31
Artery / arteriole
Vein / venule
Capillary
B
Artery / arteriole
Vein / venule
Capillary
0
20
40
60
80
100
Kirrel2-positive blood vessels (%)

Figure S10. Localization of Kirrel2 in the mouse cerebral cortex, related to Figure 7.

(A) Immunofluorescence images of the eight-week-old male mouse cerebral cortex immunostained with the indicated Abs. a, artery/arteriole; b, vein/venule; and c, capillaries. Arrowheads, arteries/arterioles; Arrows, veins/venules; *, capillaries. Scale bars, 20 μm. (B) The ratio of Kirrel2-positive blood vessels in the cerebral cortex is shown as a bar graph. These images are representative of three independent experiments.

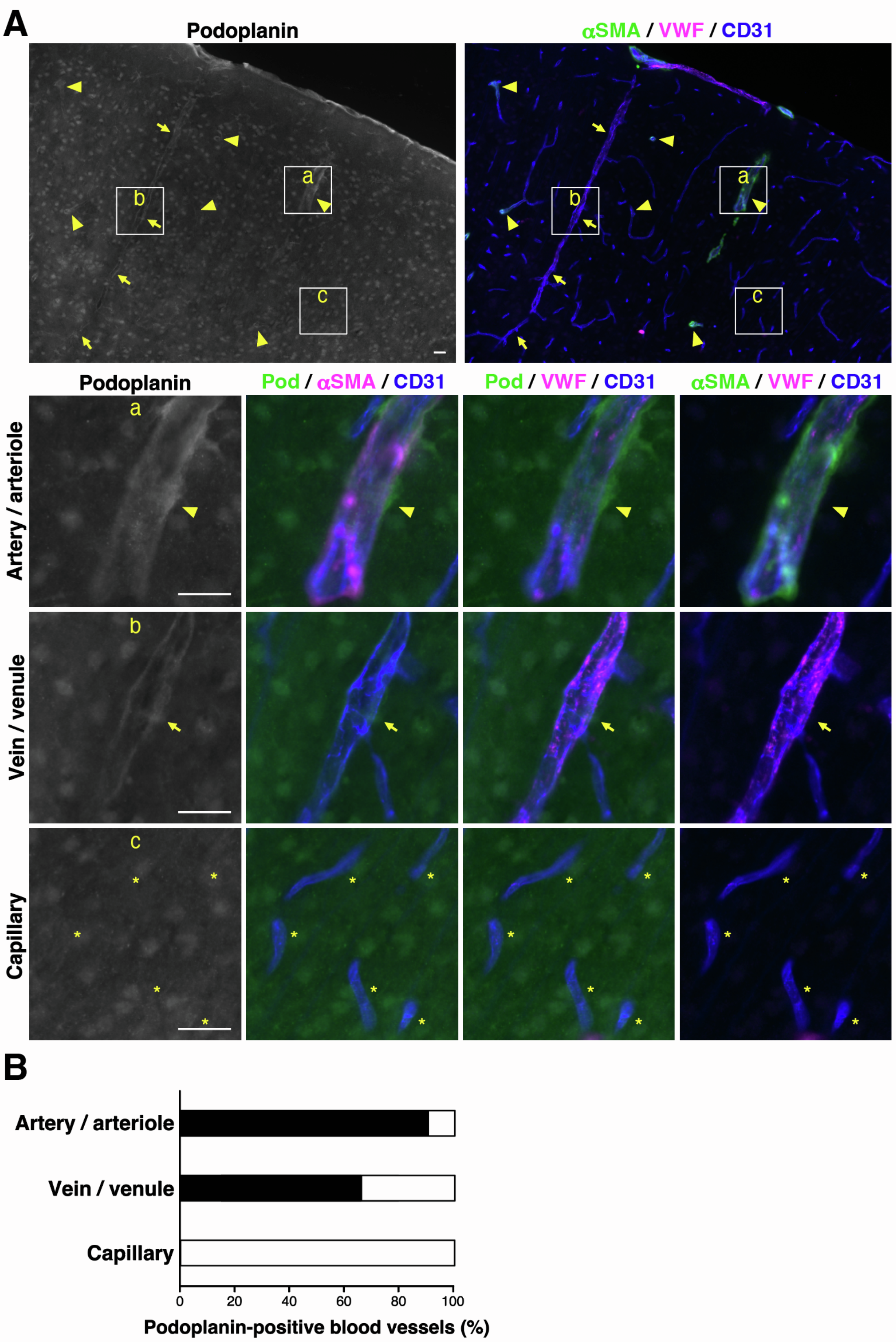
A
Podoplanin
αSMA / VWF / CD31
a
b
c
Podoplanin
Pod / αSMA / CD31
Pod / VWF / CD31
αSMA / VWF / CD31
Artery / arteriole
Vein / venule
Capillary
B
Artery / arteriole
Vein / venule
Capillary
0
20
40
60
80
100
Podoplanin-positive blood vessels (%)

Figure S11. Localization of podoplanin in the mouse cerebral cortex, related to Figure 8. Immunofluorescence images of the eight-week-old male mouse cerebral cortex immunostained with the indicated Abs. Pod, podoplanin. a, artery/arteriole; b, vein/venule; and c, capillaries. Arrowheads, arteries/arterioles; Arrows, veins/venules; *, capillaries. Scale bars, 20 μm. (B) The ratio of podoplanin-positive blood vessels in the cerebral cortex is shown as a bar graph. These images are representative of three independent experiments.

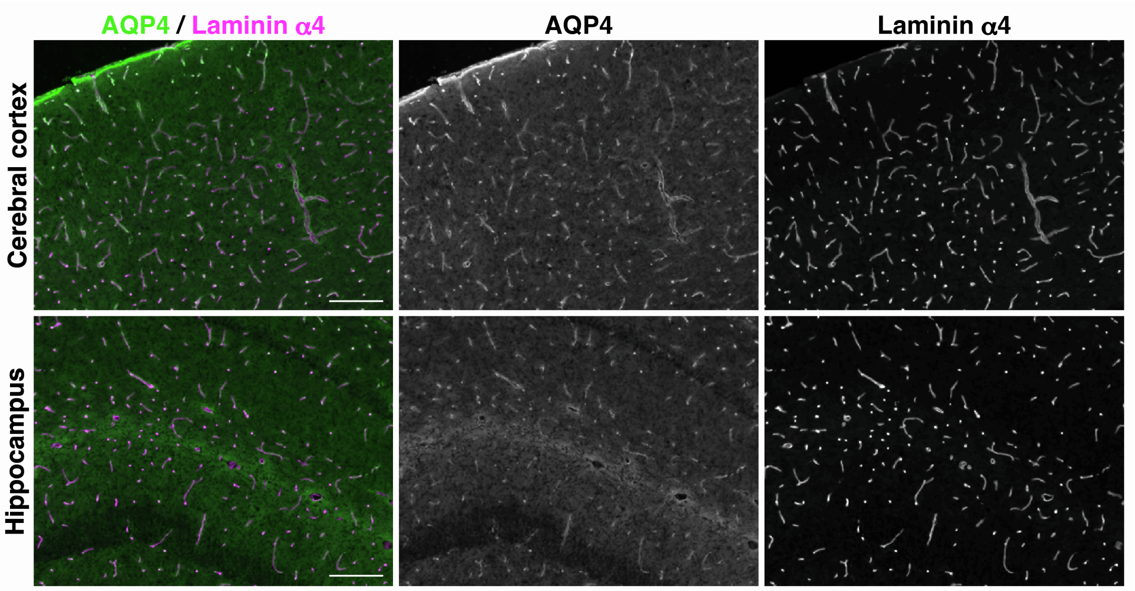

Figure S12. AQP4 expression in the mouse cerebral cortex and hippocampus, related to Figures 6–8.

Immunofluorescence images of the eight-week-old male mouse brain immunostained with the indicated Abs. Top, cerebral cortex. Bottom, hippocampus. Scale bars, 100 μm. These images are representative of three independent experiments.

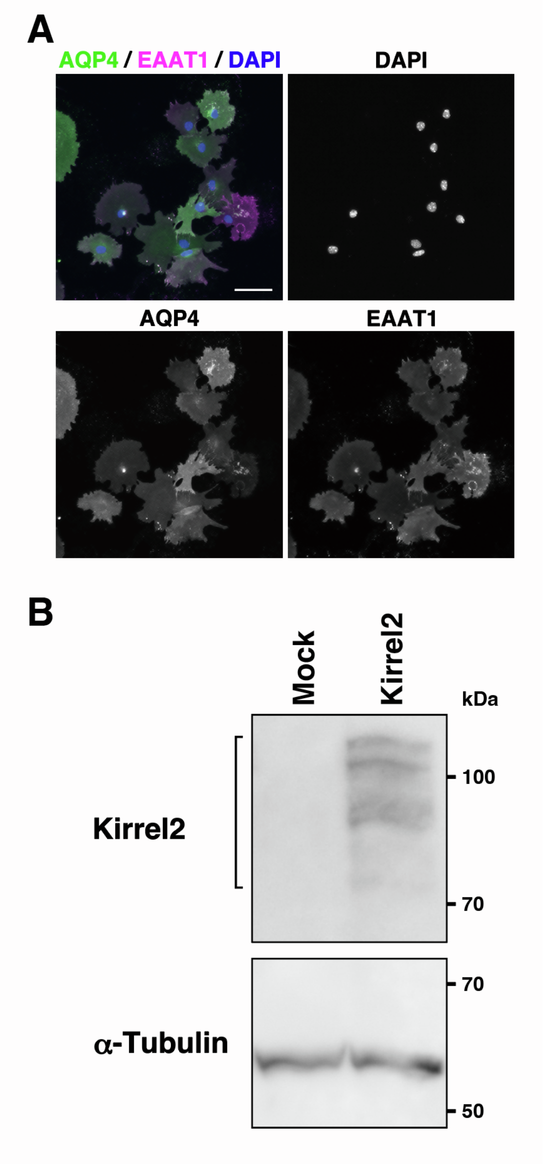

Figure S13. Kirrel2 expression in primary astrocytes, related to Figure 12.

(A) Immunofluorescence images of primary astrocytes immunostained with the indicated Abs. Scale bar, 50 μm. (B) Primary astrocytes were transfected with Kirrel2 and GFP-mem vector (Kirrel2) or control vector (Mock). At six days after transfection, cells were lysed and subjected to immunoblot analysis with the indicated Abs. Three μg of total protein was loaded in each lane. These images are representative of three independent experiments.

Supplemental references

S1. Vanlandewijck, M., He, L., Mäe, M.A., Andrae, J., Ando, K., Gaudio, F.D., Nahar, K., Lebouvier, T., Laviña, B., Gouveia, L., et al. (2018). A molecular atlas of cell types and zonation in the brain vasculature. Nature *554*, 475–480. 10.1038/nature25739.

S2. He, L., Vanlandewijck, M., Mäe, M.A., Andrae, J., Ando, K., Gaudio, F.D., Nahar, K., Lebouvier, T., Laviña, B., Gouveia, L., et al. (2018). Single-cell RNA sequencing of mouse brain and lung vascular and vessel-associated cell types. Sci. Data *5*, 180160. 10.1038/sdata.2018.160.

S3. Farhy-Tselnicker, I., Boisvert, M.M., Liu, H., Dowling, C., Erikson, G.A., Blanco-Suarez, E., Farhy, C., Shokhirev, M.N., Ecker, J.R., and Allen, N.J. (2021). Activity-dependent modulation of synapse-regulating genes in astrocytes. eLife *10*, e70514. 10.7554/elife.70514.

S4. Yosef, N., Xi, Y., and McCarty, J.H. (2020). Isolation and transcriptional characterization of mouse perivascular astrocytes. PLoS One *15*, e0240035. 10.1371/journal.pone.0240035.
